# Nuclear DNA Methylation and Chromatin Condensation Phenotypes Are Distinct Between Normally Proliferating/Aging, Rapidly Growing/Immortal, and Senescent Cells

**DOI:** 10.18632/oncotarget.942

**Published:** 2013-03-26

**Authors:** Jin Ho Oh, Arkadiusz Gertych, Jian Tajbakhsh

**Affiliations:** ^1^ Translational Cytomics Group, Department of Surgery, Cedars-Sinai Medical Center, Los Angeles, CA, USA; ^2^ Chromatin Biology Laboratory, Department of Surgery, Cedars-Sinai Medical Center, Los Angeles, CA, USA; ^3^ Bioinformatics Laboratory, Department of Surgery, Cedars-Sinai Medical Center, Los Angeles, CA, USA

**Keywords:** DNA methylation, chromatin condensation, cell proliferation, aging, senescence, cancer, 3D imaging, cell-by-cell analysis

## Abstract

This study reports on probing the utility of *in situ* chromatin texture features such as nuclear DNA methylation and chromatin condensation patterns — visualized by fluorescent staining and evaluated by dedicated three-dimensional (3D) quantitative and high-throughput cell-by-cell image analysis — in assessing the proliferative capacity, i.e. growth behavior of cells: to provide a more dynamic picture of a cell population with potential implications in basic science, cancer diagnostics/prognostics and therapeutic drug development. Two types of primary cells and four different cancer cell lines were propagated and subjected to cell-counting, flow cytometry, confocal imaging, and 3D image analysis at various points in culture. Additionally a subset of primary and cancer cells was accelerated into senescence by oxidative stress. DNA methylation and chromatin condensation levels decreased with declining doubling times when primary cells aged in culture with the lowest levels reached at the stage of proliferative senescence. In comparison, immortal cancer cells with constant but higher doubling times mostly displayed lower and constant levels of the two *in situ*-derived features. However, stress-induced senescent primary and cancer cells showed similar levels of these features compared with primary cells that had reached natural growth arrest. With regards to global DNA methylation and chromatin condensation levels, aggressively growing cancer cells seem to take an intermediate level between normally proliferating and senescent cells. Thus, normal cells apparently reach cancer-cell equivalent stages of the two parameters at some point in aging, which might challenge phenotypic distinction between these two types of cells. Companion high-resolution molecular profiling could provide information on possible underlying differences that would explain benign versus malign cell growth behaviors.

## INTRODUCTION

Cells that share the same genotype, may not necessarily present the same phenotype, including structural and functional properties that can be assayed with a plethora of existing technologies. The differences largely arise from layers of information beyond the DNA sequence, collectively termed epigenetic signatures, of which the currently most popularly investigated are differential DNA methylation, histone modifications, and micro RNA (miRNA) display. All three modalities may influence chromatin conformation in mammalian cells: both, locally for regulating site-specific gene expression and globally to modulate the plasticity of the higher-order organization of the genome [1-3]. Particularly, changes in DNA methylation patterns have been associated with the progression of cells during differentiation [4-7] as well as the etiology and pathology of complex traits [8,9]. Differences in DNA methylation patterns between normal and cancer cells have been well documented [10-12]. Cancer cells usually display hypermethylation of a relatively small number of single gene promoters mostly in gene-rich genomic regions termed CpG-islands, leading to silencing of certain tumor suppressors involved in cell-cycle regulation, DNA mismatch repair, cellular differentiation, and apoptosis. This phenomenon is often coexistent with hypomethylation at the global DNA (gDNA) level, a large portion of which occurs in repetitive elements; potentially inducing activation of latent retrotransposons with the consequence of genome-wide mutations and genome instability [13] alongside with substantial spatial reorganization of chromatin in cell nuclei, which can be visualized by light microscopy [14–18]. This differential topology can be also be triggered by exposing cells to strong demethylating agents [19,20].

Recent advancements in cellular imaging and computational image analysis have made it feasible for large volumes of images to be analyzed in relatively short amount of time at substantially lower costs, compared to genome-wide molecular assays such as DNA microarrays and massively-parallel sequencing, when being performed at the single-cell level. Even though these techniques are rapidly improving in sensitivity towards single-cell resolution and becoming gradually miniaturized and automated, still two factors will remain for a while, which attenuate their use in a cell-by-cell mode: a) the high cost accumulation, and b) the computational complexity for data interpretation. Furthermore, imaging modalities have the advantage of interrogating cells in their native environment, thus adding contextual information to subcellular structure analysis. Therefore, we need to appreciate affordable technologies that provide high statistical values and acknowledge cellular heterogeneity. With regard to that, 3D quantitative DNA methylation imaging (3D-qDMI) has been particularly developed as a tool towards the causal assessment of global DNA demethylation, heterochromatin decondensation, and relevant genome reorganization in cell nuclei to characterize cells in response to environmental changes, for directed differentiation of stem cells and therapeutic reprogramming by demethylating agents [18–24]. The effects are measured through *in situ* analysis of nuclear structures (chromatin texture) representing methylated CG-dinucleotides (MeC) and gDNA, which are delineated by fluorescence labeling and analyzed via machine-learning algorithms. 3D-qDMI is a high-throughput, imaging-based assay that allows for the rapid cell-by-cell analysis of MeC topology in thousands of individual cells, with the ability to identify and flexibly eliminate outlier cells in order to leverage data confidence.

Importantly, a connection between global hypomethylation and aging has been proposed [25], as reviewed by Pogribny and Vanyushin [26], based on original observations in the different organs of various species [27,28] and later confirmation with *in vitro* assays using organ-derived cultured cells [29–34]. Therefore, global hypomethylation can also be found in senescent cells [35–39]. Replicative senescence (RS), originally known as permanent growth arrest and repressible by pharmacological intervention [40], is a naturally occurring event in normally dividing cells after a certain number of mitotic doublings [41]. Typically, growth arrest in RS occurs during G_1_-phase of the cell cycle and is accompanied by telomere shortening [42], the expression of senescence-associated β-galactosidase (SA-β-gal) [43], and the appearance of extremely condensed genomic areas known as senescence-associated heterochromatin foci (SAHF) [44]. Additionally, senescent cells mostly display a distinct enlarged and flat cellular morphology [45].

Chromatin reorganization has been suggested as a significant contributor to aging [49–50]. Separate from RS, accelerated senescence can also be induced in cells following exposure to certain stress factors: this is more specifically referred to as stress-induced premature senescence (SIS) [51]. Physiologically aged and prematurely aged genomes also undergo wide-ranging changes in epigenetic modifications that lead to chromatin reorganization [52,53]. Early experiments in mammalian cells have demonstrated the occurrence of a global decline in DNA methylation in cultured cells including primary fibroblasts from different species compared with their immortalized counterparts [54,55]. The overall decline of methylation results mostly from the loss of DNA methylation at repetitive regions that represent about 55% of the human genome and are normally highly methylated. Age-related global hypomethylation concerns in particular satellite repeats (Sat2 and Satα DNA) as part of constitutive heterochromatin located at pericentric and centromeric loci [54–56], as well as interspersed repeat sequences such as short interspersed nuclear elements (SINES) and long interspersed nuclear elements (LINES) that have been reported to become hypomethylated during aging [57]. The importance of retrotransposons in aging was supported by recent evidences in which the class of Alu sequences — the most abundant primate SINE — was found demethylated in the context of adult stem cell aging, due to elevation in DNA damage as a result of demethylation-induced increase in Alu transcription [58]. Interestingly, adipose-derived stem cells, which undergo senescence in culture, could be rejuvenated by suppressing Alu expression: a result that challenges the original principle of the irreversibility of cellular senescence. Also, several histone modifications are affected during aging. Although driving mechanisms for chromatin and epigenetic changes during aging are currently unknown, it has been suggested that the epigenetic alterations are largely triggered by DNA damage, reviewed by Oberdoerffer and Sinclair [49]. In this scenario, randomly occurring DNA damage leads to chromatin remodeling with various functional consequences. Aged genomes are characterized by increased DNA damage and high levels of persistent DNA breaks. In particularly, and in line with the morphological changes in chromatin, changes on the histone-level can range from depletion such as in the case of histone H2A [59] and changes in the abundance level of histone-tail modifications. The heterochromatin-associated trimethylation of histone H3 lysine 9 and the transcriptionally repressive trimethylation of histone H3 lysine 27 are largely lost in aged and prematurely aged cells [60,61]. Conversely, global trimethylation of H4 lysine 20 increases with age [62]. Furthermore, probably as a consequence of loss of pericentromeric heterochromatin structure, physiologically aged and premature aged cells express normally silenced heterochromatic satellite repeats [54–56].

Investigations addressing the relationship between tumorigenesis and senescent cells, have led to earlier considerations hypothesizing that cellular senescence may act as a tumor suppressing mechanism [63–71]. Even pre-malignant lesions may have a high chance of becoming senescent cells and stop proliferation, depending on the availability and activity of certain TSGs such as *p53* or *p16*. This fact induced an initial enthusiasm for senescence as a strategy in cancer therapy. More recent experimental results have led to reconsiderations of this approach. Senescent cells, although permanently growth-inhibited in turn may drive tumor cells and even normal cells in the tumor microenvironment towards more aggressive growth behavior by promoting chronic inflammation via pro-inflammatory cytokine secretion [72,73]. Therefore, benefits or disadvantages of the presence of senescent cells may very well be context dependent. Along these lines, a hallmark of malignant epithelial cancer cells in comparison with their normal counterparts and benign cancer cells is their significantly higher proliferation rate, which they often acquire during the transformation phase in the early stages of tumorigenesis. A better understanding of these differences through studies of epigenetic features in conjunction with chromatin organization in cells may also lead to improvements in cancer diagnosis and prognosis as well as therapies.

Recently, three-dimensional DNA methylation imaging (3D-qDMI) was introduced with the notion of characterizing cells and tissues based on their nuclear global DNA methylation distribution patterns (MeC phenotypes) in a cell-by-cell mode. Applications of this technology have been utilized in the evaluation of demethylating effects of epigenetic drugs and the progression of human and mouse embryonic stem cells in early differentiation [19–24]. In here, we report on using 3D-qDMI to probe the utility of MeC-related chromatin texture features (MeC phenotypes) in characterizing cells based on their proliferative capacity, i.e. growth behavior. Being able to characterize fixed cells in conjunction with this important characteristic could provide a more dynamic picture of a cell population, therefore having tremendous implications in cancer diagnostics/prognostics and anti-cancer drug development.

## RESULTS

Additional experiments were performed, in which primary and cancer cells were synchronized in interphase to investigate any cell cycle-specific fluctuations in said features. Since the main objective of this study was to observe if changes in chromatin condensation and spatial nuclear MeC patterns can be correlated to differences in cellular proliferation capacity, it was necessary to verify that our 3-D image analysis is able to distinguish between differential nuclear texture information independent from the cycle stage of the cell.

### Differential growth behavior of cells

**Figure 1:**
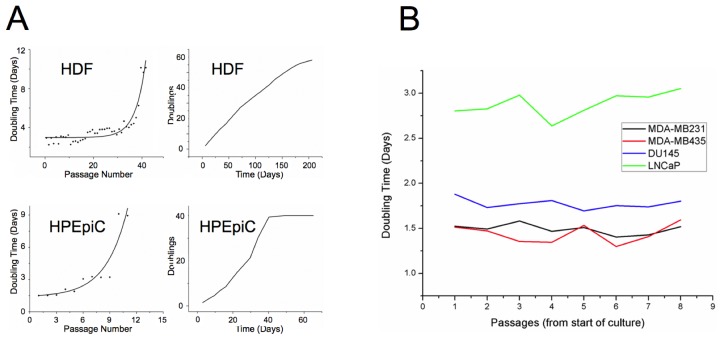
Growth rate of aging primary and cancer cells in culture (A) Doubling times are plotted against passage numbers (left panel) and cumulative doublings are plotted against total days in culture for HDF and HPEpiC cells. Prostate epithelial cells that have been established from adult tissue seem to approach senescence much earlier than neonatal dermal fibroblasts. For HDF cells, an abrupt increase in doubling times occurs around passage 40, while similar behavior can be seen for HPEpiC cells at around passage 10. (B) Cancer cells used in this study, also show variability of doubling times, with LNCaP cells growing significantly slower than the other three cell lines. However, growth rates of cancer cells do not change much with increase in passage numbers, indicating their immortality.

This study analyzed six different human cell types: including two primary cells, neonatal dermal fibroblasts (HDF) — the most extensively studied cell type in the context of cellular aging — and prostate epithelial cells (HPEpiC), as well as four human cell lines, modeling androgen-sensitive and insensitive prostate cancer (DU145 and LNCaP, respectively) and metastatic breast cancer MDA-MB-231 and MDA-MB-435, currently disputed as melanoma-derived cell line). Primary cells were grown to RS, while cancer cells were cultured in parallel to provide sufficient statistical samples by monitoring proliferation over equivalent time periods. At specific time points during culture, a fraction of cells was analyzed with 3D-qDMI. Cell counts and doubling times during culture for both types of primary cells and the four cancer cell lines used in this study are shown in Figure 1. Doubling times increased in primary cells as cells reached senescence, while doubling times stayed nearly constant for all cancer cell lines, as anticipated. We observed that DU145, MDA-MB-231 and MDA-MB-435 grew at a fairly similar rate, whereas the androgen-sensitive LNCaP cells grew relatively slower. We assume that this might have been due to lack of androgen in the culture medium. The results confirm that proliferative capacity of primary cells rapidly decrease as they age in culture, while this capacity stays nearly constant for immortal cancer cell lines.

Furthermore, in order to also assess differential growth behavior and correlated MeC phenotypes of primary and cancer cells in response to induction of accelerated senescence (SIS), cells were exposed to H_2_O_2_ following previously published protocols [51, 74, 75].

### MeC/DAPI codistribution correlates with proliferative capacity of cells

**Figure 2:**
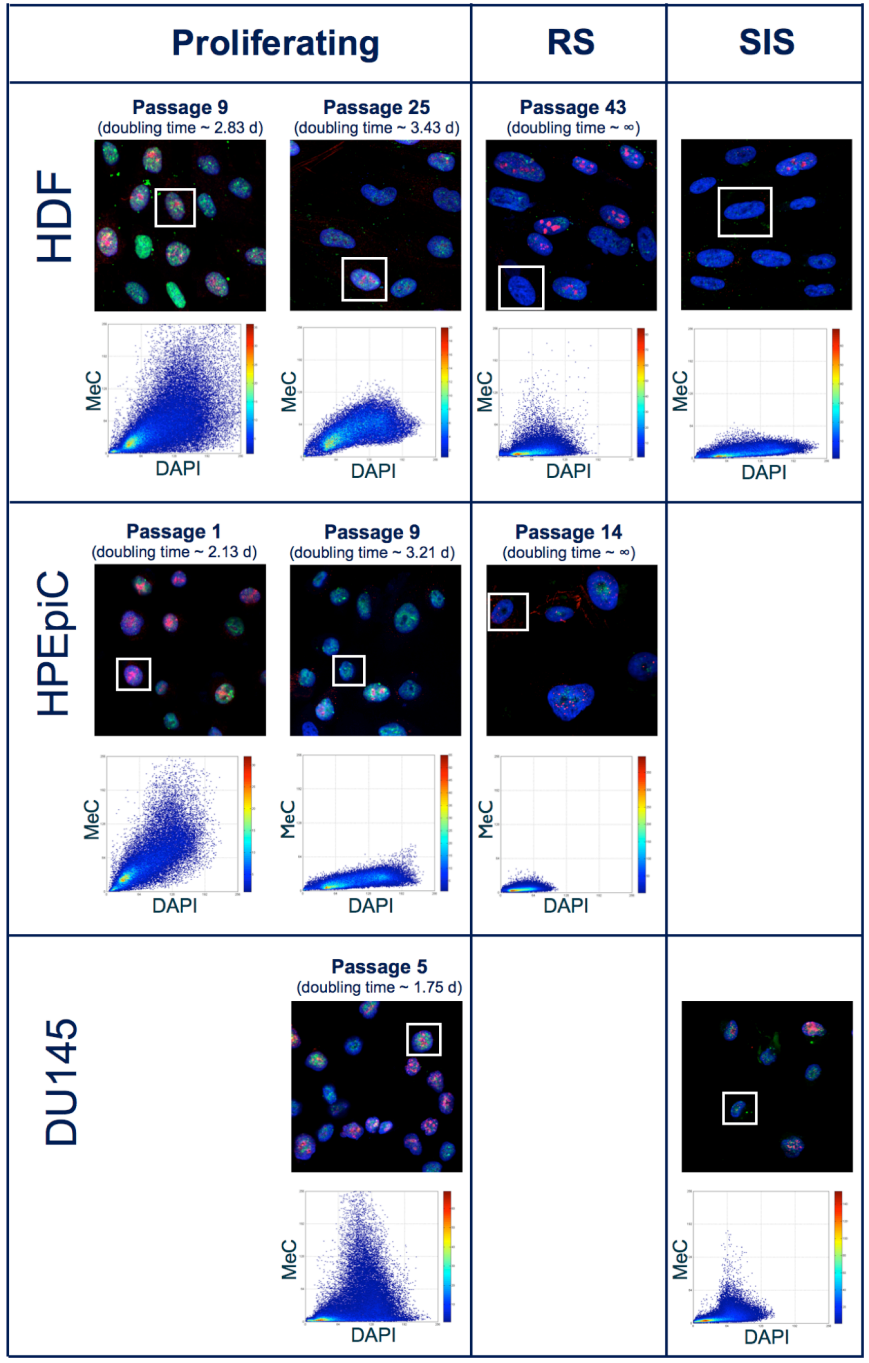
MeC/DAPI codistribution in proliferating and senescent cells Confocal mid-section images of primary and cancer cells at selected passages during culture and in response to oxidative stress (SIS): As HDF and HPEpiC cells age, there is a general decline in global MeC (green) signal. In parallel, the number of cells presenting the proliferation marker Ki-67 (red) decreases with increased passage number. Cells at higher passage numbers exhibit larger nuclei. These qualitative phenotypes, as gleaned from visual impressions, correlate with quantitative measurements: i.e. increasing doubling times and a flattening of the MeC/DAPI codistribution graphs in respective scatter plots (shown for selected framed nuclei) in aging cells. In contrast, the representative and immortal DU145 cancer cell populations comprise a majority of Ki-67-positive cells, with doubling times that are much shorter than primary cells. A convergence towards lowest MeC-signal levels can be observed in senescent populations for the two categories of cells, independent from the cause of growth arrest, either replicative or stress-induced (by H_2_O_2_ exposure).

Immunofluorescence (IF) samples were generated every 3-5 passages for primary cells, while for cancer cells, at least 5 different IF samples were created at selected passages throughout the culturing process. In addition to visualization of MeC and gDNA, we also co-stained for the proliferation marker Ki-67. Although it is possible to quantify Ki-67 signal intensities using the 3D-qDMI framework, for the purposes of this study Ki-67 was only used as a qualitative marker to roughly distinguish between proliferating and declined cells. Typically, greater than thousand nuclear regions of interest (ROI) were recognized in most analyzed IF samples. Of these nuclear ROIs, those categorized as “dissimilar” and, thus identified as outliers — based on methods previously described in [21,22] — were excluded from subsequent image analysis. In all images used in this study, the percentages of nuclear ROIs deemed as outliers were less than 1%, and their exclusion did not significantly alter data confidence. Each nuclear ROI is analyzed individually using 3D-qDMI, and the cell-specific data are then pooled to form population-level statistics. This study focused on four categories of analytical parameters to delineate DNA methylation and chromatin condensation patterns in each nucleus: (1) physical information, particularly the spatial dimensions (in *x*-, *y*-, *z*-coordinates) and volume of ROI; (2) MeC/DAPI codistribution; (3) statistics for a subset of signals, i.e. percentage of low-intensity MeC (*LIM%*) and low-intensity DAPI (*LID%*) pixels within ROIs, determined by a threshold on image-inherent features; and (4) topological voxel-level parameters, which represent degrees of *meth* and *cond*, and the correlation of the two at the voxel level as *assoc*. Figure 2 depicts qualitative changes of DNA methylation patterns in cultured primary cells based on the first three types of analytical parameters: as cells age, the relative pixel intensities in MeC and Ki-67 channels decrease, while the nuclear volume, delineated by DAPI counter-staining, increases. These changes correlate with increasing doubling times. Higher-passage populations comprise a majority of enlarged senescent cells. In our study, we also chemically induced growth arrest in aggressively proliferating prostate cancer cells (DU145) and a subset of HDF by exposing cells to H_2_O_2_ as previously mentioned. The stress-induced senescent cancer cells analogously showed a differential MeC/DAPI pattern in comparison with their proliferating counterparts, although the latter as usual showed lower global MeC levels than normal cells. Interestingly, there was no significant difference in the MeC/DAPI patterns between replicative senescence and stress-induced senescence within the same cell type and also between HDF and DU145 cells.

### Topological voxel analysis for a more dynamic analysis of nuclear textures

To investigate the correlation between MeC topology/nuclear texture and cell proliferation a new analytical module called topological voxel analysis (TVA) was developed to extend pre-existing analytical modules in 3D-qDMI [21,22]. In our previous work with 3D-qDMI, we focused on the analysis of topological distribution of low-intensity MeC (LIM) and low-intensity DAPI (LID) sites within the nuclear region of interest (ROI) to highlight changes in chromatin density between drug-treated and untreated cells. We experienced a more differential analysis at the nuclear periphery, which contain mostly facultative heterochromatin [19,22]. However, differences more in the interior of nuclei, which preferentially are composed of euchromatin and constitutive heterochromatin, were more difficult to discern via this approach, and requested a more careful selection of signal cut-off levels for determining LIMs and LIDs. In order to better utilize chromatin condensation as a nuclear texture feature, we applied TVA that is based on a different sub-compartmentalization of the nucleus than previously used for the study of LIM/LID topology.

To quantify differences in the spatial codistribution of DAPI and MeC signals the three parameters *cond*, *meth*, and *assoc* were calculated for each nuclear ROI. Our approach was inspired by previous publications delineating heterogeneity and condensation levels of stained chromatin for 2-D images [76–80]. Chromatin condensation in a given voxel is measured by the parameter *cond*, representing the condensation level of genomic DNA, while the relative degree of methylation in the same given voxel is measured by the parameter *meth*. The parameter *assoc* describes the correlation of low- or high-intensity pixels in both channels to determine how associated one signal in a given physical space is with the other signal in the same space. Once these parameters are found for each voxel within the nuclear ROI, they can be processed by two different methods. First, topological changes in parametric value can be measured at each normalized position from the nuclear center to the periphery, and subsequently these trend lines for each nuclear ROI in a cell population can be compared to determine how much topological variation in chromatin condensation and methylome distribution exists for a given population of cells. Second, volume-weighted mean of voxel-level parametric values are determined to represent a singular parametric value for the whole nuclear ROI. Detailed methodology and explanation of how analyses using the voxel-level parameter occur are presented in the Supplementary Materials (File 5).

### No significant differences exist between DNA methylation and chromatin condensation patterns of cells at different stages of the cell cycle

**Figure 3:**
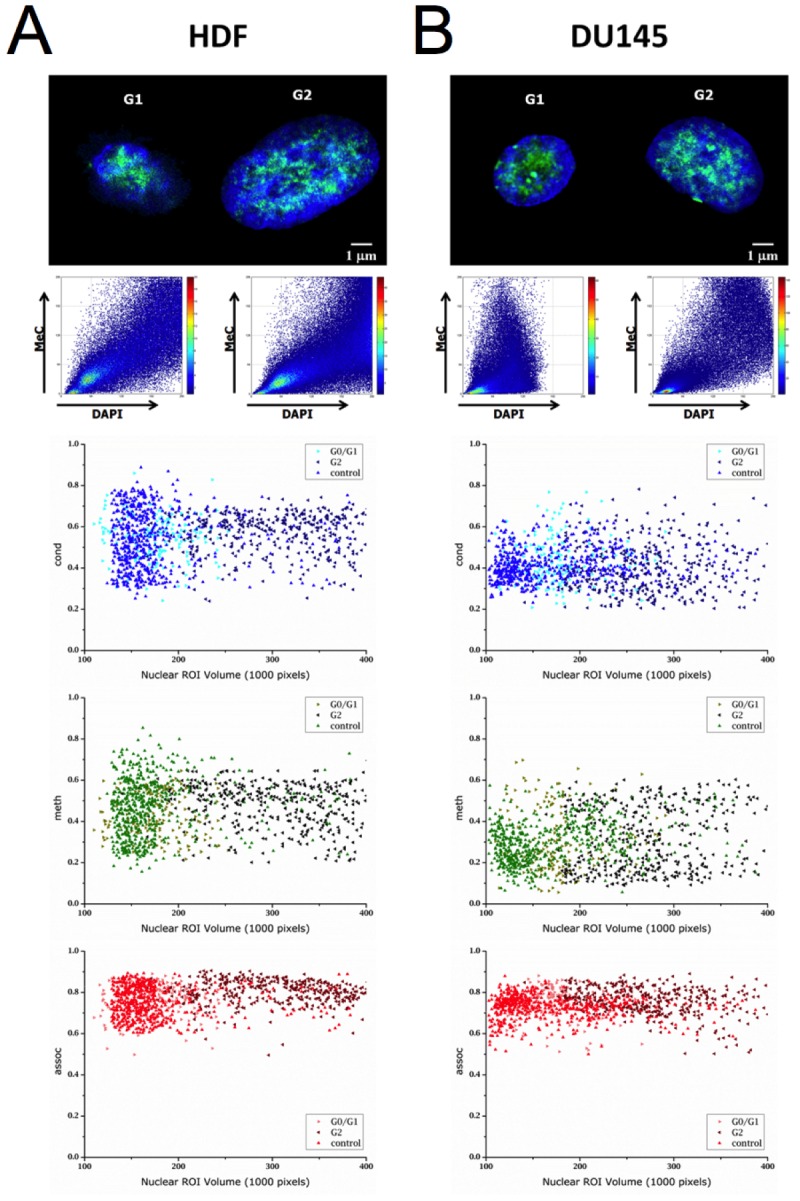
Voxel-based parameters are independent from cell cycle stages Confocal mid-section images of HDF (A) and DU145 cells (B) are shown with their respective MeC/DAPI codistribution scatter plots. Below the images are graphs displaying the cell type-specific respective distribution of topological voxel analysis parameters *cond*, *meth*, and *assoc* at the two major interphases of the cell cycle (G_0_/G_1_ and G_2_) and for related unsynchronized control populations. In all graphs, each data point represents one nuclear ROI volume. The parameter distributions display a high similarity across the three cell groups.

To ensure that spatial MeC and global DNA co-distribution patterns do not vary significantly during the interphase cell cycle, as to adversely affect the data confidence of 3D-qDMI analysis, 3D-qDMI with topological voxel analysis was performed on three types of populations for both, HDF and DU145 cells: G_0_/G_1_-enriched and G_2_-enriched populations, together with an unsynchronized control population. Synchronization was performed using a combination of a double-thymidine block and serum starvation, and cell population synchrony was verified by fluorescence-activated cell sorting (FACS) based on propidium iodide (PI) staining. For all enrichment assays, a majority (>75%) of cells was found to be in the targeted phase. Furthermore, since serum starvation was used to arrest cells in G_0_/G_1_ phase, viability of synchronized populations was tested: as >90% of cells being viable the cell enrichment step was successful without significant induction of cell death. More details on verification of cell cycle synchronization and cell viability can be found in the Supplementary Materials (File 1). Figure 3 displays examples of G_0_/G_1_ and G_2_ synchronized cells with distributions of topological voxel analysis parameters (*cond*, *meth*, and *assoc*) (Table 1) plotted against the volume of nuclear ROIs. As can be seen from this distribution scatter plot, there are no statistically significant differences in the values of each parameter for any of the three groups of cells; in other words, DNA methylation and relevant chromatin condensation patterns of cells are not unique to a particular stage of the cell cycle. Table 1 shows the mean and standard deviation for various 3D-qDMI analysis parameters. The only significant difference between the three populations — for each cell type — is the nuclear (ROI) volume, which is reasonable, considering the diploid state of cells in G_2_ versus G_1_ due to the extra copy of genomic DNA that was synthesized during the intermediate S phase of the cell cycle. From these results, we conclude that the image-analysis features utilized to discriminate cells in growth behavior are detached from cell cycle phases. Therefore, feature data collected from cells that are randomly fixed in non-synchronous populations will not be influenced by this inherent parameter of living cells.

**Table 1:** Voxel-based parameter values for HDF and DU145 cells at different cell cycle phases

HDF G0/G1	HDF G2	HDFcontrol	Analysis Parameters (μ ± σ)	DU145 G0/G1	DU145 G2	DU145 control
560	783	1066	Total cells analyzed	412	504	625
176 ± 33	306 ± 63	172 ± 48	Nuclear ROI volume (1000 pixels)	174 ± 40	283 ± 67	171 ± 59
48.2 ± 14.1	51.1 ± 15.4	50.4 ± 12.5	LID%	51.7 ± 14.4	54.2 ± 15.0	51.4 ± 17.8
36.9 ± 20.0	35.7 ± 26.0	39.8 ± 21.0	LIM%	50.9 ± 19.2	54.8 ± 23.7	51.8 ± 24.2
0.55 ± 0.10	0.57 ± 0.10	0.53 ± 0.14	cond	0.43 ± 0.11	0.40 ± 0.12	0.42 ± 0.08
0.43 ± 0.10	0.48 ± 0.11	0.46 ± 0.14	meth	0.31 ± 0.15	0.32 ± 0.15	0.30 ± 0.10
0.76 ± 0.08	0.80 ± 0.06	0.75 ± 0.08	assoc	0.75 ± 0.08	0.76 ± 0.07	0.73 ± 0.06

### Chromatin demethylation and decondensation occur with increased passage numbers in primary cells

**Figure 4:**
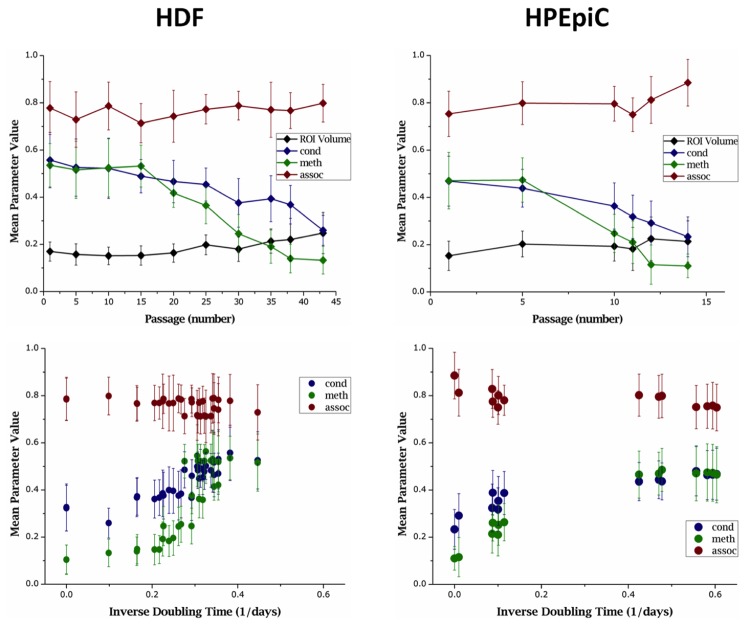
Averaged parameter values of primary cell populations at different stages in culture Change in nuclear ROI volume (represented in million pixels) and parameters *cond*, *meth*, and *assoc* are shown against both, passage number (top panel) and inverse of doubling time (bottom panel). With each successive passage, both HDF and HPEpiC show decrease in *meth* and *cond*. These changes correspond to the cells' abating proliferation capacity, as measured by doubling times.

We then assessed the fourth type of analytical parameter in the same samples as above. For that, the three TVA features *meth*, *cond*, and *assoc*, were measured in all cell types at specific time points within the entire culture duration for HDF and HPEpiC cells (Figure 4). As previously shown in Figure 1, the two primary cell types reached RS after around 50 and 15 doublings, respectively. Table 2 presents a tabular form of data given in Figure 4 for selected passages. The entire data set is available in the Supplementary Materials (File 2). Prior to reaching RS, we observed an increase in doubling times for these cells with successive passage, as a sign of decline of the overall proliferative capacity of these cells. This decline was generally accompanied by a decrease in Ki-67 presentation, as evidenced in Figure 2.

**Table 2:** Voxel-based parameter values for HDF and HPEpiC at selected passages

HDF P10	HDF P25	HDF P43	Analysis Parameters(μ ± σ)	HPEpiC P1	HPEpiC P5	HPEpiC P14
1407	1283	514	Total cells analyzed	1449	1668	680
2.82	3.42	10.14	Doubling Time (days)	1.47	2.11	infinity
151 ± 35	194 ± 41	248 ± 87	Nuclear ROI volume (1000 pixels)	150 ± 61	201 ± 55	214 ± 87
1675 ± 522	1689 ± 299	1191 ± 250	DAPI raw intensity (max value: 4096)	1657 ± 456	1516 ± 315	939 ± 386
1353 ± 566	1009 ± 223	577 ± 284	MeC raw intensity (max value: 4096)	1149 ± 379	1311 ± 369	332 ± 291
51.0 ± 12.3	54.9 ± 13.7	80.7 ± 7.8	LID%	48.0 ± 18.6	56.4 ± 16.6	84.6 ± 11.7
51.5 ± 10.5	54.5 ± 12.3	81.1 ± 11.7	LIM%	46.7 ± 20.0	51.6 ± 14.3	87.4 ± 10.1
0.53 ± 0.13	0.46 ± 0.07	0.26 ± 0.06	cond	0.46 ± 0.10	0.44 ± 0.08	0.23 ± 0.08
0.52 ± 0.13	0.36 ± 0.08	0.13 ± 0.06	meth	0.47 ± 0.12	0.46 ± 0.10	0.11 ± 0.05
0.78 ± 0.10	0.77 ± 0.06	0.80 ± 0.08	assoc	0.75 ± 0.09	0.80 ± 0.09	0.89 ± 0.10

**Figure 5:**
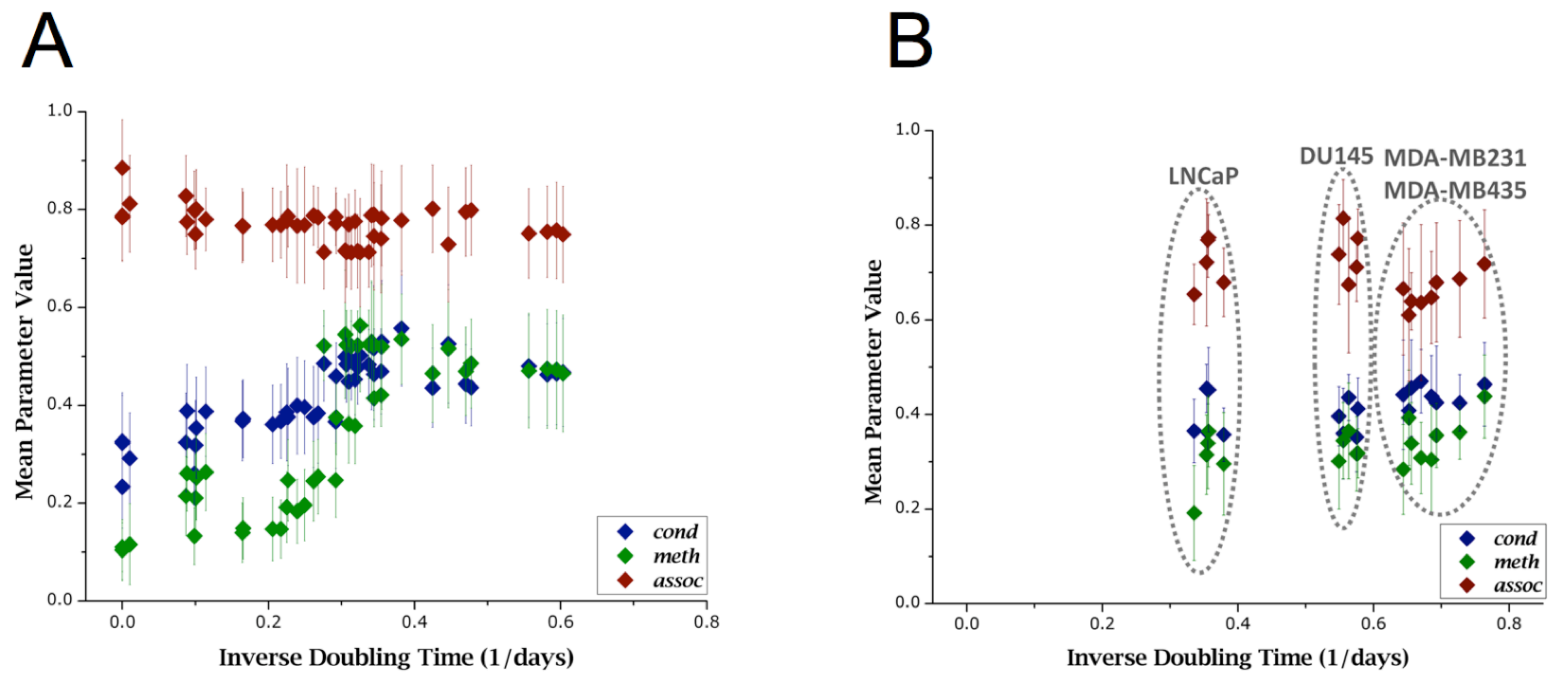
Voxel-based parameters in primary and cancer cells (A) The graph presents pooled data from all primary cells. A distinct positive correlation can be seen in *meth* and *cond* compared to inverse of doubling times. Low parameter values at low growth capacity (as measured by doubling times) correspond to seemingly senescent cells, and high parameter values correspond to early passage cells with short doubling times; with overall high correlativity to doubling times; *meth*: r^2^ = 0.772, *cond*: r^2^ = 0.757, and *assoc*: r^2^ = 0.410. We interpret that DNA demethylation along with chromatin decondensation increase in primary cells as cell growth slows down. (B) In comparison: according to pooled data from all cancer cells, immortal cells of all four kinds show a fairly equally low *meth* value (global hypomethylation) at significantly higher proliferation rates (as measured by doubling times). Even though there are differences in doubling times amongst the cancer cell lines, no significant correlation of parameter values with doubling times was measured (*cond*: r^2^ = 0.441; *meth*: r^2^ = 0.474; *assoc*: r^2^ = 0.369).

Through each successive passage, both HDF and HPEpiC show a progressive loss of global methylation and became highly hypomethylated upon reaching their respective RS point, as measured by an increase in *LIM%* and a decrease in the parameter *meth*. Global hypomethylation becomes highly evident after passage 30 in HDF cells and passage 10 in HPEpiC cells. At the same time, an increase in *LID%* and a decrease in *cond* could be observed, implying that global hypomethylation is accompanied by chromatin reorganization in aging cells. Therefore, as expected, the parameter *assoc*, which measures the correlation of low- or high-intensity pixels in both DAPI and MeC channels at a given voxel cube, also increased as cells RS. This increase can be attributed to the overall increase in the distribution of pixels within the ROI that are of both, low-intensity DAPI and low-intensity MeC. These changes in global DNA methylation and spatial genome organization are concordant with visual impressions provided by high-resolution images of stained cells, as previously shown in Figure 2. When the correlation of the analytical parameters to doubling times from all images of primary cell IF-samples was calculated, parameters *cond* and *meth* showed high levels of correlation with doubling times (Figure 5A).

### Hypomethylation is evident in cancer cells compared with primary cells

As expected, TVA features did not change much for specimens collected at various times in the culture of cancer cells. Table 3 shows the averaged results from these specimens for each cell type.

**Table 3:** Voxel-based parameter values for cancer cells at selected passages

Analysis Parameter	DU145	LNCaP	MDA-MB231	MDA-MB435
Doubling time (days)	1.77	2.81	1.50	1.42
Total cells analyzed	2966	2992	3902	4753
Nuclear ROI Volume (103 pixels) (μ ± σ)	156 ± 57	183 ± 54	129 ± 43	136 ± 41
LID% (μ ± σ)	62.5 ± 12.2	39.4 ± 13.2	4.76 ± 12.6	45.6 ± 13.9
LIM% (μ ± σ)	67.7 ± 9.6	63.7 ± 17.1	61.3 ± 17.4	50.9 ± 23.9
cond (μ ± σ)	0.319 ± 0.098	0.398 ± 0.092	0.439 ± 0.135	0.443 ± 0.111
meth (μ ± σ)	0.301 ± 0.117	0.301 ± 0.131	0.336 ± 0.134	0.360 ± 0.109
assoc (μ ± σ)	0.742 ± 0.093	0.720 ± 0.080	0.688 ± 0.178	0.633 ± 0.116

Overall, cancer cells showed lower degrees of methylation, as evidenced by high *LIM%* and low *meth*, compared to primary cells with similar proliferation rates. Primary cells with higher proliferation capacities (in the earlier passages) usually showed higher degrees of global methylation with *meth* ~ 0.5, as previously shown in Figure 4, while for cancer cells, values of *meth* remained well below this level for all cancer cell types. On the other hand values for *LID%* and *cond* were similar to that of highly proliferative, early passage primary cells. As in cancer cells, global hypomethylation was not necessarily correlated with signs of chromatin decondensation. In these types of cells we consistently registered *assoc*-values lower than those measured in primary cells; although this difference was not large enough to be statistically significant. Therefore, in order to further search for measureable chromatin texture-based differences between highly proliferative primary and cancer cells, we calculated the degree of correlation between the TVA parameters and the cells' doubling. Figure 5B shows a scatter plot of doubling times and 3D-qDMI analytical parameters for each cancer cell line used in this study. Unlike in primary cells, none of the TVA parameters correlated satisfactorily with doubling times in cancer cell lines. This is an interesting difference observed between normal and cancer cells, which can be interpreted as such: values of these parameters may be disconnected from growth rates (and associated proliferative capacity) in transformed cells.

To determine how well TVA parameters correlate to doubling times, Pearson's correlation coefficients were calculated between each parameter and the inverse of doubling times, as shown in Table 4.

**Table 4:** Pearson's correlation coefficients of parameter values compared to inverse of doubling times for primary and cancer cells

	cond	meth	assoc
Primary Cells	0.757	0.772	0.410
Cancer Cells	0.441	0.474	0.369

**Figure 6:**
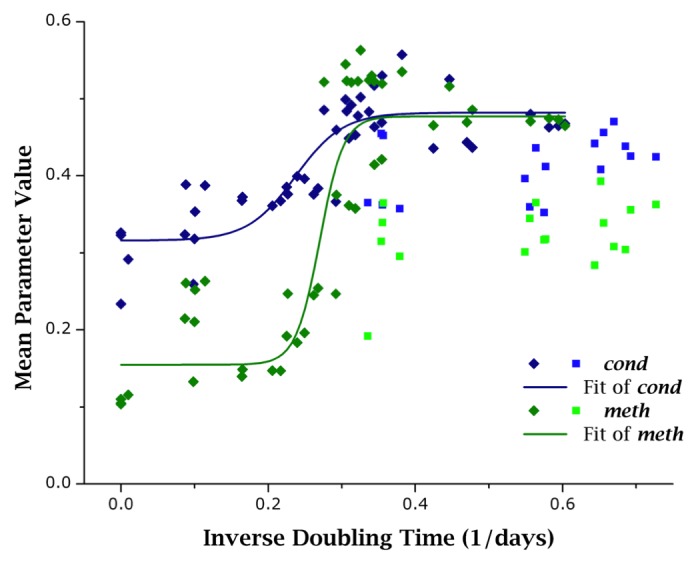
Different growth behaviors of primary cells and cancer cells as evidenced by voxel-based parameters When comparing primary cell data (dark, diamond markers) to cancer cell data (light, square markers), it can be seen that while primary cells correlate well to growth capacity (as measured by doubling time), cancer cells do not. In this figure, both *cond* and *meth* are fit with a logistic curve, which was determined to be the best fitting curve based on F-test results. Parametric values from cancer cell populations lie further away from fitted curves and from primary cell populations.

In primary cells, both *cond* and *meth* showed good level of correlativity with *r2* > 0.75. The same was not experienced for cancer cells: instead both showed poor correlativity with *r2* < 0.5. Using this knowledge, primary cell data was fitted to a logistic curve. Several models were explored, including exponential, logarithmic, polynomial, and linear models. The logistic model was chosen from ANOVA (analysis of variance) analysis. Figure 6 exhibits the same data as in Figures 5A and 5B overlaid with logistic fitting curves. From these results it is apparent that cancer cells display parametric values outside the confidence range that can be estimated from primary cell data, and these differences could be used to discriminate primary and cancer cells. However, this claim needs to be verified in future follow-up studies.

### DNA methylation and chromatin condensation patterns of replicative senescent cells and stress-induced senescent cells are similar

In order to establish a correlative relationship between proliferative capacity of cells — as measured by cellular growth rates — and TVA features, it was necessary to set a baseline value for these analytical parameters using a known non-proliferative cell. As mentioned above, primary cells were grown until they reached replicative senescence, in order to obtain cells that lack growth potential. Separate from RS, stress-induced premature senescence was studied by treating HDF and DU145 cells with hydrogen peroxide (H_2_O_2_). Details on concentration and time of peroxide treatment and pre- and post-treatment cell counts are available in the Supplementary Materials (File 3). Senescence was verified in both, RS and SIS cells by two methods in parallel subcultures at same propagation time points: (i) a subset of cells was simultaneously immunostained for the proliferation marker Ki-67 in the IF, and a marked decrease in the presence of Ki-67 signals in both types of senescent cells over regularly proliferating cells was noticed; (ii) another subset of cells was colorimetrically stained for presence of senescence-associated β-galactosidase (SA-βgal). RS and SIS cell populations presented an absolute majority of large and flat cells with strong SA-βgal expression compared with populations with predominantly proliferating cells (data not shown).

As previously demonstrated in Figure 2, structural differences between normally proliferating cells and senescent cells could be retrieved by visual inspection, while no significant differences were observed between RS and SIS cells. For primary cells, H_2_O_2_-treatment yielded typical MeC phenotypes that were very similar to MeC phenotypes of RS cells. Both types of growth-arrested cells have strongly reduced MeC loads and the associated TVA features verify these changes quantitatively. Table 5 shows image analysis results for untreated proliferating (control), as well as RS- and SIS-HDF cells. Both visual and quantitative data reveal no difference in the two types of senescent cells regarding said features.

**Table 5:** Voxel-based parameter values for proliferating, replicative senescent, and stress-induced senescent HDF cells

Analysis Parameter	Normally Proliferating	Replicative Senescence	Stress-induced Senescence
Total cells analyzed	1066	1482	1323
Nuclear ROI Volume (103 pixels) (μ ± σ)	142 ± 47	215 ± 81	189 ± 56
LID% (μ ± σ)	0.544 ± 0.047	0.792 ± 0.064	0.753 ± 0.144
LIM% (μ ± σ)	0.505 ± 0.133	0.792 ± 0.133	0.773 ± 0.118
cond (μ ± σ)	0.471 ± 0.077	0.280 ± 0.078	0.316 ± 0.128
meth (μ ± σ)	0.479 ± 0.160	0.162 ± 0.092	0.102 ± 0.095
assoc (μ ± σ)	0.722 ± 0.079	0.816 ± 0.092	0.790 ± 0.093

**Figure 7:**
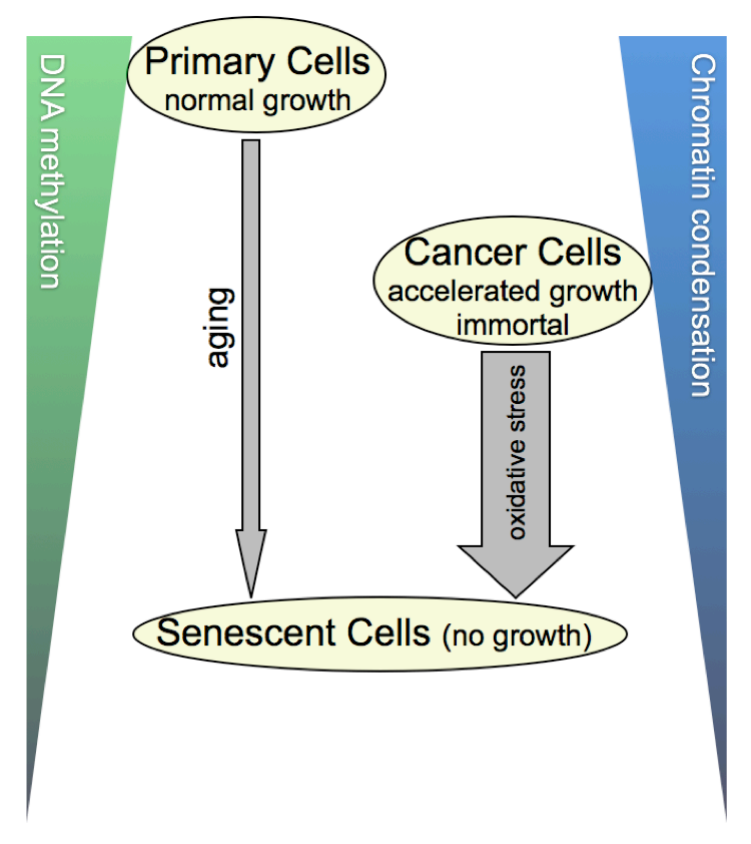
Cellular DNA methylation and concurrent chromatin condensation levels The illustrated scheme is derived from the 3-D image- and flow-cytometrical results of our cell-by-cell study, summarizing the correlations we found. In a nutshell, cancer cells with an accelerated growth behavior compared with their normal counterparts take a meta-stage in global DNA methylation and chromatin condensation levels between the latter and senescent cells that have reached growth arrest. As benign primary cells seem to reach cancer-cell equivalent stages of the two parameters at some point in aging, this calls the question as to whether the underlying sequence of molecular profiles may differ between the two categories of cells. Along the same lines of thought, it would be informative exploring this matter also for SIS, which is depicted as a shorter but more forceful process (thick arrow) than gradual aging (thin arrow).

In the case of cancer cells, such as DU145, SIS-type cells exhibited: (i) larger nuclear ROI, (ii) an increase in *LID%* and *LIM%*, and (iii) decreased values for *cond* and *meth*. Thus, the LIM/LID and the *meth/cond* data sets are in agreement with one another. Table 6 compares image analysis results for untreated proliferating and SIS-DU145 cells. The parametric (feature) values from nuclei in the latter group are on par with those observed in RS primary cells, indicating that there is also no difference between transformed (originally immortal) cells and primary cells, when they are driven or accelerated into senescence. Therefore, it seems as if the stage of growth arrest is correlated with the differential TVA features, independent from the cells original growth character and the method by which senescence was elicited. In a closer look at our measures, we gleaned that untreated cancer cells exhibit lower degrees of *meth/cond* (~30% less) concordant with higher values for LIM/LID (~21%) than early passage primary cells. Therefore, concerning these features, aggressively growing cancer cells seem to take an intermediate level between normally proliferating and senescent cells, as illustrated in Figure 7.

**Table 6:** Voxel-based parameter values for proliferating and stress-induced senescent DU145 cells

Analysis Parameter	Proliferating	Senescence
Total cells analyzed	534	426
Nuclear ROI Volume (103 pixels) (μ ± σ)	166 ± 58	213 ± 66
LID% (μ ± σ)	0.661 ± 0.088	0.749 ± 0.158
LIM% (μ ± σ)	0.666 ± 0.101	0.823 ± 0.096
cond (μ ± σ)	0.353 ± 0.092	0.259 ± 0.128
meth (μ ± σ)	0.318 ± 0.073	0.179 ± 0.077
assoc (μ ± σ)	0.773 ± 0.062	0.799 ± 0.079

## DISCUSSION

The main objective of this study was to investigate the relationship between the spatial nuclear distribution of methylcytosine — as an epigenomic landmark in conjunction with chromatin condensation patterns — and cellular growth behavior, using a novel image-cytometrical approach. This study was inspired by the notion to discover chromatin texture-based features that can be extracted by automated image analysis for generating a more dynamic and activity-based picture of individual cells in a population; with conceivable applications in pre-clinical drug testing and cancer pathology. By delineating and quantifying *in situ* spatial distributions of genomic DNA and MeC residues, 3D-qDMI delivers a dynamic and motion-based picture of cells. Especially in clinical pathology, where traditional staining methods provide a more static picture reminiscent of the tissue's history but mostly challenging the prediction of tissue (and disease) fate, extracted TVA features could support tissue characterization towards more confident prognosis. Thus, 3D-qDMI may contribute as a high-throughput, and cost-effective companion assay for screening of tumors and other epigenetics-related abnormalities. Our approach follows the appeal of exploiting differential DNA methylation patterns as potentially powerful epigenetic markers in the characterization and therapy cancer malignancies [81–83]. Changes in the DNA methylation profile of cancer cells are often comprised of hypermethylation of single gene promoters, leading to aberrant silencing of disease-related genes, and global hypomethylation of repetitive sequences in intergenic regions and heterochromatic areas of the genome: tumor cells harbor at average 20–45% less MeC content compared with their normal counterparts [13, 84–89]. The effect of hypomethylation on cancer cells has been evidenced as correlating with chromosomal instability [90,91]. Though some variations exist in the DNA methylation profile among different types of tumors, the general trends of specific hypermethylation, mostly within CpG islands, and global hypomethylation appear to be ubiquitous in all tumors [36, 92]. Therefore, a secondary objective of this study was to assess the feasibility of distinguishing and/or discriminating cells with different proliferative capacities, based on their spatial nuclear DNA methylation phenotypes and relevant higher-order genome organization.

The most important consideration behind any imaging-based assay is to accurately quantify imaged signals and *in situ* patterns at or even beyond the level of human visual perception. In our previous publications [19, 21,22], we had introduced image-analysis tools to highlight differences in DNA methylation patterns based on MeC/DAPI colocalization patterns and 3-D maps of LIMs and LIDs. The latter feature required a careful selection of defined intensity thresholds, which works well for the more consistent structure of the nuclear periphery, but may face challenges in the mapping of more subtle changes in the more internal nuclear regions: due to dynamic local differences in the euchromatin and heterochromatin portions of DNA. Therefore, we have extended 3D-qDMI with a new analytical module, termed topological voxel analysis. The idea behind this amendment is that fluctuations in chromatin reorganization, that in our case accompany DNA demethylation, can be traced through the measurement of DAPI intensity changes in the nucleus.

When using 3D-qDMI to track changes in *in situ* DNA methylation and chromatin condensation patterns, an inquiry we made was, whether our approach would be sensitive to differences in MeC/DAPI codistribution patterns based on the unsynchronized cells being at different interphase cell cycle phases. This consideration is important in dissecting pattern changes that are attributed to conditional alterations (of interest) from cell cycle-specific influences. In our analytical practice, cells in M- (mitotic) phase were excluded from assessment for two reasons: (a) most of these cells go through different nucleation phases and do not allow for a proper ROI determination (based on DAPI counterstaining), (b) because the unique and highly compact chromatin conformation is not suitable for differential pattern recognition. Because of these properties, all M-phase cells were automatically flagged as outlier cells by 3D-qDMI software and excluded from further analysis, based on criteria given in [21]. It is also important to note that the number of M-phase cells, in a given specimen, compared to the number of cells in the interphase is relatively small, and thus their elimination from topological analysis will not have an impact on the final statistical outcome.

Although trying to interpret chromatin condensation using microscopy is not a new approach [48–52], to the best of our knowledge, our approach is novel in retrieving and cross correlating changes in both signatures — DNA methylation texture patterns and chromatin organization — with cellular proliferation, by means of confocal microscopy and comprehensive 3D image analysis. This was based on the hypothesis that higher resolved and therefore more differential texture information would be extractable from 3D analysis than from 2D analysis or maximum intensity projections of fluorescence signals. Additionally, we have chosen cell types and conditions that would allow us to associate the two signatures with the three major groups of cell growth behavior: normally proliferating primary cells, highly proliferating cancer cells, and minimally proliferating senescent (or near-senescent) cells derived from these two cell types. The latter group enabled us to capture nuclear signatures in a more continuous form during which primary cells gradually changed their capability from growing at their fastest (normal) rate in the early passages all the way to irreversible growth stop. Our results show that 3D-qDMI enhanced with the newly developed analytical methods is able to quantify phenotypic differences visualized through optical imaging, and that these differences match previously reported descriptions of epigenetic phenomena in cancer cells and senescent cells.

Initially, this study had planned to include a G_1_-only synchronization using thymidine block and nocodazole, which arrests cells in the mitotic phase. However, one major technical difficulty arose when using this approach. Cells arrested in the M-phase usually rounded up and detached from the glass surface; these detached cells could not be strongly re-attached (to allow immunofluorescence staining) to the substrate before cells progressed from G_1_ to S- phase. Furthermore, although not the primary reason for not using nocodazole, a recent publication from Cooper *et al*. [93] has suggested that nocodazole cannot synchronize cells properly. Therefore, acknowledging technical limitations in fixed cell assays and the limitations with nocodazole-inhibition, arresting cells to quiescence (G_0_ phase) via serum starvation seemed to be a reasonable alternative. While G_0_-phase cells are temporarily non-dividing, they are not post-mitotic cells and could be triggered to enter the cell cycle under appropriate conditions [94]. Most importantly, methylation patterns of G_0_/G_1_ synchronized populations were not affected by deleterious drug treatment.

Flow cytometry of G_0_/G_1_ and G_2_ synchronized populations for both HDF and DU145 cells revealed that the cell cycle synchronization was highly efficient, as the vast majority of cells were found to be in the targeted cell cycle phase. At the same time, the image-cytometrical analysis of respective parallel populations using 3D-qDMI showed high degrees of homogeneity regarding DAPI/MeC codistribution patterns, further supporting the results from flow cytometry. Using 3D-qDMI, no statistically significant differences were found between the G_0_/G_1_ and G_2_ synchronized populations and the control population for both HDF and DU145 cells, in all topological analysis criteria (*LID%*, *LIM%*, *cond*, *meth*, and *assoc*). This result may not be surprising considering that methylation of hemi-methylated DNA (replicated strand) by DNA methyltransferase *Dnmt1* is tightly coupled with the replication process [95, 96]. Since this process is meant to mirror the molecular methylation patterns on the replicated strand, it should not change the distribution of methylcytosine within the genome and thus MeC/DAPI codistribution should essentially stay constant. This is in line with the fact that chromatin organization should be conserved during S-phase and inherited to daughter cells upon cell division [94, 97,98]. Therefore the differential methylation phenotypes we observed with 3D-qDMI in this study can be robustly associated with growth behavior and are not skewed by the inherent cell-cycle variability within analyzed cell populations. This fact renders DNA methylation phenotyping highly valuable for pathological tissue characterization in which synchronicity of cells within their native environment is naturally not the case.

With this confidence, the relationship between cellular proliferation capacity and chromatin organization was studied. Primary cells were grown to their Hayflick limit, as they reached replicative senescence. The number of cells in culture was counted after every passage to generate a comprehensive database of growth rates. The proliferation capacity (as measured by doubling time) was fairly consistent for over the first 30 passages in HDF cells and the first 9 passages in HPEpiC cells. Past this point, the doubling times slowly increased followed by a sudden leap around passage 40 for HDF cells and passage 11 for HPEpiC cells. This event coincided with the majority of cells becoming senescent, and a residual minimal growth for a few cells within the propagated populations. The RS-cells showed a significant level of global hypomethylation, and enlarged nuclear morphology, and these changes could be detected by light microscopy and more thoroughly quantified with 3D-qDMI. To better understand topological changes in DNA methylation and accompanying chromatin reorganization, 3D-qDMI considers a variety of analytical parameters. Although these parameters are all related to the codistribution of MeC/DAPI signals found in the same image, they do not behave in exactly the same fashion. For example, in HDF cells, *meth* begins to decline significantly around passage 25, but this behavior is not seen by change in *LIM%*, although these two values are correlated. These differences can be used in creating a unique picture of the analyzed cells. When cells became essentially post-mitotic, reorganization of genomic DNA was observed. This is evidenced by an increase in *LID%* and the correlated decrease in *cond*. However, at the same time, we observed a gain in the nuclear ROI volume. As, the total nuclear content of the cells did not change over time, a reduction in gDNA condensation could be associated with two phenomena: (i) global hypomethylation, and (ii) an increase in nuclear space. In comparison, the growth characteristics of cancer cells remained fairly consistent throughout the culture. A fraction of cells were fixed for immunofluorescence assay and subsequent image analysis; for each cancer cell type, at least five different cell populations (collected from different time points) were analyzed for this comprehensive study. 3D-qDMI parameters revealed that all cells showed reduced MeC pixel intensities, compared with primary cells. Global DNA hypomethylation in cancer cells is a well-known phenomenon, and is in stark contrast to that of normally proliferating primary cells at early passages, whereas both types of cells exhibited quite similar spatial intensity distributions in the DAPI channel.

Prior to any regression analysis, it was necessary to set a baseline value for non-proliferating cells. For this purpose, we decided to model this behavior with senescent cells. Representative for cancer cells, DU145 cells were treated with H_2_O_2_ to pressure cells into stress-induced senescence. Regarding the analytical parameters used in this study, values for *cond* and *meth* decreased, suggesting that SIS exerts a dual effect: DNA demethylation and concurrent chromatin decondensation. In parallel, HDF cells were also treated with H_2_O_2_ to observe if SIS yields values different from RS cells. However, no difference could be observed between the two types of senescent cells by 3D-qDMI. This observation leads to the conclusion that: untreated cancer cells, which generally exhibited lower degrees of meth/cond concordant with higher values for LIM/LID than early passage primary cells, seem to take an intermediate level between normally proliferating and senescent primary cells (Figure 7). In other words, the stage of senescence is correlated with values of the TVA-derived chromatin features that are below the level of cancer cells. However, a decrease in global DNA methylation and an increase of DNA decondensation both have causal associations with genomic instability, and the degree of both phenomena has so far been correlated with the degree of malignancy. Thus, the data suggests that by pushing the global level of the two cell properties below a certain level, the cells independent of their proliferative capacity and genomic make-up, could be driven into senescence. It would be interesting to identify the underlying DNA sequences and their genomic localization that match or even contribute to these particular cell behavior characteristics. This would provide insight as to whether there is a specific order of demethylation and structural changes taking place on the genome when the cells are driven towards senescence, and whether there are even differences if this process occurs gradually or more forcefully, also depending on the genomic and epigenomic make-up of the naïve cells.

Correlation analysis of voxel-based parameters to growth rates demonstrated distinct differences between primary and cancer cells. In primary cells, the parameters *LID%*, *LIM%*, *cond*, and *meth* showed high levels of correlativity to doubling times, whereas similar trends could not be found in cancer cells, even though the four cancer cell lines showed different doubling times that span a similar range observed with primary cells during *in vitro* aging. Statistical analysis with two-sample t-test revealed that these differences were significant (p < 0.01). Thus, it is conceivable that the relationship between growth rate and voxel-based parameters could be used to discriminate between normally growing cells, such as primary cells, and aggressively fast-growing cells, such as cancer cells. For application in cancer diagnostics, ideally, any phenotyping should be based only on image-intrinsic features, and not dependent on doubling times or other growth-related statistics, which constitute *a priori* knowledge that would not be available in fixed biopsy sections. Nevertheless, this type of approach could become useful in live-cell assays, which allow for monitoring of cells such as in pre-clinical drug screening and personalized treatment development with cultured patient cells; especially in the development of epigenetic drugs that aim at remodeling cells and their growth characteristics.

In conclusion, this study verifies the capability of 3D-qDMI to map and quantitate dynamic changes in DNA methylation patterns and associated chromatin organization. Using this cell-by-cell imaging-based assay together with novel informatics tools we were able to make two key observations. 1) We could reconcile, a gradual increase in global hypomethylation and decondensation of genomic DNA for primary cells during aging in culture, confirming previous reports based on molecular assays [29–34]. In comparison, cancer cells of various origins, although having different doubling times did not display any differences in the two nuclear phenomena, and revealed constant proliferation rates over the entire culturing time. The results are in agreement with previous documentations [49,54,99]. 2) Cells, independent from their class and original growth capacity, converge to the highest level of global DNA hypomethylation and decondensation when reaching senescence. This fact concurs with significant changes to higher-order genome organization in our study, as previously reported in conjunction with age-related DNA damage [100]. Recent evidences suggest that these alterations of the chromatin structure is caused by the relocation of chromatin modifiers such as histone deacetylase SIRT1 [101] and the nucleosome remodeling and histone deacetylase (NuRD) complex in mammals [102] and from repetitive DNA in constitutive heterochromatin as a result of DNA damage due to an impaired DNA repair machinery. Based on the abovementioned differences, we were able to determine a correlation between the reciprocal analytical parameters *LIM%/LID%* and *meth/cond* and the proliferative capacity of cells. As both, aging and transforming cells exhibit overlapping changes in their methylation profiles, the relocation of epigenetic machinery from its normal distribution in the genome could be a link between heterochromatin demethylation and the hypermethylation of cancer-related genes, leading to coexistence of the two aberrations in aging and cancer cells as discussed by Teschendorff et al. in [103]. Therefore, it is becoming increasingly obvious that cell growth behavior is tightly related to the interplay between the various epigenetic mechanisms and spatial genome organization. Combined imaging- and sequencing-based methylation structure analyses with recently available technologies [104–107] could unravel the differential methylation patterns and related genome supraorganization in aging cells and improve our understanding of how these signatures are distinct in cellular fate: namely between growth deceleration towards senescence and transformation into aggressively proliferating cancer cells, with implications in drug-based therapy.

## MATERIALS AND METHODS

### Cell culture

Six types of human cells were used in this study: dermal fibroblasts (HDF), and prostate epithelial cells (HPEpiC) as primary cells (both ScienCell, Carlsbad, CA), the androgen-sensitive human prostate cancer cell line LNCaP and the androgen-insensitive prostate cancer cell DU145, as well as the breast cancer cell line MDA-MB231 and MDA-MB435 of controversial origin [108] (all ATCC, Manassas, VA). Cells were cultured at 37°C and 5% CO_2_ following standard culture procedures. HPEpiCs were grown in Prostate Epithelial Cell Basal Medium (ScienCell) supplemented with Prostate Epithelial Cell Growth Kit (ScienCell). LNCaP cells were grown in RPMI 1640 medium (Mediatech, Manassas, VA, USA) supplemented with 10% fetal bovine serum (FBS) (Mediatech), 1% Penicillin-Streptomycin (Mediatech), and 2.5 μg/mL Fungizone (Life Technologies, Carlsbad, CA). All other cells (HDF, DU145, MDA-MB231, and MDA-MB435) were grown in Dulbecco's Modification of Eagle's Medium (DMEM) (Mediatech) supplemented with 10% FBS, 1% Penicillin-Streptomycin, and 2.5 μg/mL Fungizone. Primary cells were grown until their RS was reached. Cancer cells were grown for 8–10 passages, so that at least 5 distinct samples could be generated. All cells were passaged at 75–80% confluency with 0.05% trypsin-EDTA (Mediatech) to maintain uniform culturing conditions. Typically, during this study, cells were usually passaged every 4-5 days. If cells were at reduced proliferation levels, as in those cells near RS, and did not reach the aforementioned confluence levels for passaging, culture medium was changed once a week. Cells were regularly monitored for both phenotype and cell counts; phenotype was evaluated by light microscopy and cell counting was performed at time of each passage with a hemocytometer, and Trypan blue staining (Invitrogen) was used to exclude dead cells from propagation statistics.

### Stress-induced premature senescence

Stress-induced premature senescence was induced in HDF and DU145 cells via application of H_2_O_2_ following previously published protocols [46,47, 70]. HDF cells used for SIS study were at passage 20. Prior to application of H_2_O_2_, cells were grown to near-confluency (~100%). For cell counting purposes, a fraction of these cells was seeded in 6-well culture plates at 8·10^5^ cells/ml, 24 hours before treatment with H_2_O_2_. SIS cells were studied at three different conditions: (i) control group (no H_2_O_2_); (ii) 200 μM H_2_O_2_, previously deemed as the appropriate level for induction of senescence [109]; and (iii) 500 μM H_2_O_2_, a lethal dose of peroxide. For each condition, confluent cells were placed in a medium containing DMEM supplemented with 1% FBS and the appropriate concentration of HPLC-grade H_2_O_2_ solution (Sigma, St. Louis, MO) for 2 hours at 37°C and 5% CO_2_. After peroxide treatment, cells grown in tissue culture flasks were detached by treatment with 0.05% trypsin-EDTA, seeded onto glass coverslips, and allowed to recover for 24 hours in regular culturing medium (DMEM supplemented with 10% FBS). Subsequently, cells were washed with phosphate-buffered saline (PBS) and fixed for immunofluorescence assay. Analogously, for SIS cells in 6-well culture plates, cells were allowed to recover for up to 7 days in regular medium. Cells in the control group and the two H_2_O_2_-treated groups were counted at three time points: (i) immediately after peroxide treatment, (ii) at 24 hours post treatment, and (iii) one week after senescence induction. Dead cells were excluded from cell counts with Trypan blue exclusion assay. Senescence in these cells were verified by light-microscopic observation of morphology, as well as staining for β-galactosidase expression with the Senescence β-Galactosidase Staining Kit (Cell Signaling Technology, Danvers, MA), as previously published [110] and according to vendor protocol.

### Cell cycle synchronization and cell viability

HDF and DU145 cells were seeded onto glass coverslips at the concentration of 10^5^ cells/mL for immunocytochemical staining and subsequent imaging via confocal microscopy. For FACS, a parallel set of cultures at the same concentration was maintained in TC-grade culture flasks, and processed as previously described [19]. Enrichment efficiency was evaluated by flow cytometry using a Becton-Dickinson FACScan (BD Biosciences). Excitation was achieved with a 488 nm laser line, and emission was collected at 617 nm. FACS data was then analyzed using ModFit LT (Verity Software House, Topsham, ME, USA). Separately, cell viability was tested, as the synchronization steps involved serum starvation. The two populations were tested for apoptosis using the Annexin V-FITC Apoptosis Detection Kit I (BD Biosciences) and flow cytometry. The results were analyzed by FACSDiva (BD Biosciences).

### Immunofluorescence staining

At set time points, a fraction of cultured cells was seeded onto glass coverslips and allowed to attach for 24 hours. Cells are then fixed in 4% paraformaldehyde/PBS, and sequentially stained with a mouse monoclonal anti-MeC primary antibody (clone 33D3, GeneTex, Irvine, CA), and an Alexa 488-conjugated goat anti-mouse polyclonal secondary antibody (Invitrogen). For some samples, cells were simultaneously stained with an additional set of a rabbit polyclonal anti-Ki-67 primary antibody (ab833, Abcam, Cambridge, MA) and subsequently Alexa 568-conjugated and a goat anti-rabbit polyclonal IgG (Life Technologies) to verify proliferation [111–113]. Although signals for Ki-67 can be quantified using methods described in the following subsection, for the purposes of this research, Ki-67 was only used as a qualitative proliferation marker. Following immunocytochemical staining, specimens were counterstained with DAPI (Invitrogen) for delineation of nuclear gDNA, as previously described [19,23].

### Image acquisition

Stained samples were imaged using an inverted confocal laser-scanning microscope (Leica Microsystems TCS SP5X Supercontinuum, Mannheim, Germany), equipped with a white-light laser for continuous excitation between 470 to 670 nm, and a 405 nm diode laser line for excitation of DAPI fluorescence. Images are acquired as a 3-D image (*x*, *y*, *z*) by collecting serial 2-D optical sections (*x*, *y*) at 250 nm increments (*z*) using a Plan-Apo 63X 1.3 glycerol immersion lens (pinhole size was 1.0 Airy unit). Each optical section is acquired at a resolution of 2048 × 2048 pixels, with a respective voxel size of 120 nm × 120 nm × 250 nm (*x*, *y*, and *z* axes), and at a dynamic range of 12 bits per pixel. As such, the field of view in each section corresponded to a 246 μm by 246 μm square. As the cell monolayer area was much larger than the field of view, the entire IF sample was tiled and serial sections were generated at each position represented by tiling. To avoid possible bleed-through of signals (cross-talk effect) between the channels, images for each channel were acquired sequentially. To improve signal-to-noise ratio, each recorded image was averaged four times. Raw images were obtained as Leica Image Format (lif) and offline-converted to a series of TIFFs for downstream image analysis.

### Computational image analysis

Acquired images were analyzed using dedicated software developed with Matlab (MathWorks, Natick, MA, USA). Parts of this analytical tool were previously reported with more detailed explanation of methods used for 3-D image segmentation [21,22, 114]. Briefly, acquired 3-D images were segmented and binarized by a threshold intensity that was determined from image-inherent features, to obtain seeds, which were used to define distinct 3-D nuclear ROIs via a modified version of seeded watershed algorithm [115] extended to three dimensions. Once nuclear ROIs are clearly defined, nuclear similarity and population homogeneity is assessed using Kullback-Leibler (K-L) divergence [116] for each cell in a given population through the extraction of colocalized MeC/DAPI patterns, allowing for identification and elimination (if necessary) of outlier cells in a given population. For all analyses used in this study, we have eliminated all nuclear ROIs deemed “dissimilar” (i.e., nuclear ROI with K-L divergence value greater than 4.5) according to definitions provided in [21]. Within defined nuclear ROIs, the chromatin condensation level and associated spatial distribution of MeC residues are analyzed by dividing a given ROI into cubes of constant voxel sizes (500 nm per side), which was decided by the physical limitations of the imaging system, namely due to the excitation wavelengths of fluorophores and the objective lens used for confocal microscopy. The methodology and justification for dividing nuclear ROI into these voxels are explained in the Supplementary Materials (File 4). The distribution and location of low- and high-intensity pixels are analyzed for each voxel. These results are later combined for further analyses by comparing the condensation patterns for DAPI signals with associated changes of methylation patterns in the MeC channel. Finally, three other types of data, which were extracted from these images, were considered in the analyses presented in this study. First, the nuclear ROI volume is defined as the number of pixels within a given ROI. Since all imaging used in this study was done at identical image settings, each pixel represents a physical space of 120 nm (*x*) × 120 nm (*y*) × 250 nm (*z*). For convenience, all nuclear ROI volumes presented in here are defined as numbers of individual pixels. Second, for both DAPI and MeC channels, the mean of pixel intensities within a defined nuclear ROI was measured. Third, based upon a threshold derived from image-inherent features, the percentage of low-intensity pixels in either DAPI (*LID%*) or MeC (*LIM%*) channel was measured and mapped in each nuclear ROI. More details on how a threshold intensity level is determined for each image is provided in the Supplementary Materials (File 6).

### Statistical analysis

Analysis of data obtained with 3D-qDMI was performed with either Matlab or Origin (OriginLab Corp., Northampton, MA). Pearson's correlation coefficients were calculated to determine the correlativity of parametric values to growth rates, while two-way ANOVA tests were applied for determining the best fitting model from primary cell data.

### Supplementary materials

The following additional files are available Supplementary Materials. File 1 shows data for verification of cell cycle synchrony with PI staining and viability test with an apoptosis screening kit. File 2 lists the values for selected 3D-qDMI parameters time point sampled during the culture of primary cells. File 3 shows the cell survival rates after induction of stress-induced senescence in HDF and DU145 cells after exposure to H_2_O_2_ at varying concentrations. File 4 describes the resolution of 3D-qDMI and how this information was exploited to determine the minimum analysis voxel cube. File 5 explains in detail the topological voxel analysis parameters introduced in this study. File 6 describes how thresholding intensity was obtained from image-inherent cues and how this process was applied to all analyses detailed in this report.

## Supplementary Tables and Figures


