# Supplementary material for: Nuclear DNA Methylation and Chromatin Condensation Phenotypes Are Distinct Between Normally Proliferating/Aging, Rapidly Growing/Immortal, and Senescent Cells
Source: Oncotarget. 2013 Mar 26;4(3):474–93. doi: 10.18632/oncotarget.942 (PMC3717309; doi:10.18632/oncotarget.942)
Supplement: Supplementary file 1 [file oncotarget-04-474-s001.pdf]

Nuclear DNA Methylation and Chromatin Condensation Phenotypes Are Distinct Between Normally Proliferating/Aging, Rapidly Growing/Immortal, and Senescent Cells- Ho Oh et al

1. Cell cycle synchronization

Cell cycle synchronization results (as analyzed by ModFit LT) are shown in Table S1.1. To maintain consistency, G_2/G_1 ratio was maintained at 1.9–2 in all populations. In all conditions, a supermajority (>75%) of cells was found to be in the desired phase. These results suggest that HDF and DU145 cells were correctly synchronized as expected.

Population type	% in G_0/G_1 phase	% in S phase	% in G_2/M phase
**HDF G_0/G_1**	79.9%	20.1%	0.0%
**HDF G_2**	0.0%	13.8%	86.2%
**DU145 G_0/G_1**	76.0%	18.0%	6.0%
**DU145 G_2**	0.0%	20.1%	79.9%

Table S1.1: Cell cycle synchronization results.

HDF and DU145 cells were synchronized to G_0/G_1 phase by serum starvation and G_2 phase by double thymidine block. After synchronization, cells were labeled with propidium iodide and subjected to flow cytometry. The FACS results were analyzed with ModFit LT.

2. Cell viability

The viability of synchronized population was tested using FITC Annexin V Apoptosis Detection Kit I (Table S1.2). Viable and not apoptotic cells ($FITC^-$, PI^-) represented ~ 90% of G_0/G_1 synchronized population and ~ 96% of G_2 synchronized population for both HDF and DU145 cells. As expected from the serum starvation treatment used in G_0/G_1 synchronization, the proportion of likely necrotic cells ($FITC^+$, PI^+) were greater in G_0/G_1 population (4.9% HDF,

5.6% DU145) than G_2 population (1.9% HDF, 1.6% DU145). However, the proportion of dead cells is minimal and should not affect the subsequent analysis.

Population type	% viable and not apoptotic ($FITC^-$, PI^-)	% likely apoptotic ($FITC^+$ PI^-)	% likely necrotic ($FITC^+$, PI^+)	% other ($FITC^-$, PI^+)
**HDF G_0/G_1**	90.3%	2.8%	4.9%	2.0%
**HDF G_2**	96.6%	0.8%	1.9%	0.7%
**DU145 G_0/G_1**	90.4%	3.8%	5.6%	0.2%
**DU145 G_2**	96.4%	1.9%	1.6%	0.1%

Table S1.2: Cell viability results.

HDF and DU145 cells synchronized to G_0/G_1 and G_2 phases were tested for viability using FITC Annexin V Apoptosis Detection Kit I. The viable and not apoptotic population (FITC Annexin V and PI negative) was around 90% in G_0/G_1 synchronized populations and around 96% in G_2 synchronized populations.

Supplementary File 2

Cell Type - Passage #	Doubling Time (days)	ROI Volume mean (pixels)	ROI Volume st_dev (pixels)	raw DAPI intensity mean	raw DAPI intensity st_dev	raw MeC intensity mean	raw MeC intensity st_dev	LID% mean	LID% st_dev	LIM% mean	LIM% st_dev	*cond* mean	*cond* st_dev	*meth* mean	*meth* st_dev	*assoc* mean	*assoc* st_dev
HPEpiC-P1	1.42	152317	63117	1685	466	1142	389	46.09%	19.04%	47.88%	19.53%	0.4675	0.1102	0.4649	0.1191	0.7494	0.0986
HPEpiC-P1	1.47	150494	60697	1657	456	1149	379	47.96%	18.61%	46.73%	20.03%	0.4628	0.1039	0.4746	0.1215	0.7547	0.0936
HPEpiC-P1	1.44	152427	59370	1665	463	1129	398	46.36%	19.18%	47.68%	20.14%	0.4647	0.1040	0.4728	0.1208	0.7576	0.0988
HPEpiC-P1	1.52	156999	65622	1704	463	1136	382	45.18%	19.50%	48.00%	19.28%	0.4799	0.1045	0.4708	0.1171	0.7514	0.0917
HPEpiC-P5	1.90	204635	55474	1511	324	1318	366	56.69%	16.78%	50.35%	14.15%	0.4365	0.0785	0.4856	0.0908	0.7990	0.0921
HPEpiC-P5	1.92	202671	53126	1544	318	1300	363	55.83%	16.75%	51.08%	14.15%	0.4435	0.0802	0.4695	0.0906	0.7953	0.0905
HPEpiC-P5	2.11	201096	54510	1516	315	1311	369	56.39%	16.60%	51.59%	14.34%	0.4357	0.0810	0.4653	0.0998	0.8021	0.0893
HPEpiC-P10	8.75	197715	62816	1535	377	793	301	71.68%	16.42%	70.36%	13.18%	0.3874	0.0911	0.2634	0.0786	0.7802	0.0639
HPEpiC-P10	11.32	192488	63677	1538	385	795	292	71.37%	16.69%	70.43%	13.22%	0.3887	0.0945	0.2609	0.0767	0.7749	0.0664
HPEpiC-P10	9.91	193032	63230	1447	401	766	355	75.01%	16.68%	72.05%	15.06%	0.3537	0.1036	0.2521	0.0866	0.8009	0.0809
HPEpiC-P10	11.49	189228	58003	1367	404	665	310	78.05%	16.20%	76.69%	13.72%	0.3240	0.1010	0.2147	0.0813	0.8279	0.0828
HPEpiC-P11	10.00	181943	91092	1316	310	603	162	66.43%	19.41%	70.60%	13.98%	0.3183	0.0916	0.2106	0.0895	0.7500	0.0710
HPEpiC-P12	100.00	224884	92238	1245	360	393	221	57.93%	17.24%	61.46%	16.63%	0.2917	0.0930	0.1158	0.0828	0.8121	0.0988
HPEpiC-P14	infinity	213894	87271	939	386	332	291	84.55%	11.69%	87.37%	10.07%	0.2336	0.0843	0.1102	0.0499	0.8853	0.0987
HDF-P1	2.62	169705	40521	1732	336	1512	525	44.07%	16.00%	47.48%	16.99%	0.5573	0.1174	0.5351	0.0919	0.7781	0.1121
HDF-P5	2.24	157468	45301	1698	349	1346	282	48.49%	16.18%	50.65%	17.82%	0.5255	0.1210	0.5161	0.1208	0.7289	0.1172
HDF-P10	2.91	148899	37665	1650	459	1337	510	52.40%	12.51%	52.01%	9.03%	0.5171	0.1276	0.5228	0.1179	0.7898	0.1022
HDF-P10	2.82	151080	34563	1675	522	1353	566	50.98%	12.28%	51.51%	10.49%	0.5300	0.1251	0.5197	0.1279	0.7821	0.0969
HDF-P10	2.93	154283	39537	1668	502	1459	578	51.92%	14.07%	51.74%	10.10%	0.5206	0.1294	0.5301	0.1237	0.7886	0.1050
HDF-P15	3.28	149423	40485	1793	285	1487	628	44.99%	16.60%	40.76%	23.39%	0.4993	0.0553	0.5450	0.1313	0.7163	0.1058

Supplementary File 2

HDF-P15	3.07	147446	42388	1807	300	1567	646	44.24%	17.08%	38.64%	23.84%	0.5021	0.0577	0.5631	0.1354	0.7124	0.1107
HDF-P15	2.97	155977	42028	1791	360	1400	291	44.40%	20.97%	43.07%	15.11%	0.4831	0.0716	0.5243	0.0699	0.7131	0.0712
HDF-P15	3.10	152995	39078	1791	367	1378	288	44.36%	20.37%	44.45%	14.71%	0.4778	0.0752	0.5228	0.0700	0.7148	0.0749
HDF-P15	3.62	155435	41477	1799	345	1388	285	44.80%	19.82%	43.39%	15.03%	0.4854	0.0764	0.5219	0.0716	0.7131	0.0751
HDF-P15	3.26	156313	41261	1808	362	1404	275	43.79%	20.06%	42.55%	14.48%	0.4839	0.0774	0.5233	0.0702	0.7133	0.0718
HDF-P15	3.19	152988	38882	1801	354	1380	281	45.12%	20.34%	43.68%	15.35%	0.4917	0.0803	0.5214	0.0727	0.7121	0.0747
HDF-P20	2.82	162922	38551	1512	430	1305	362	56.23%	16.15%	50.07%	19.97%	0.4692	0.0870	0.4212	0.0640	0.7402	0.1108
HDF-P20	2.90	164423	38855	1509	411	1300	352	56.36%	16.33%	50.38%	20.11%	0.4634	0.0925	0.4146	0.0584	0.7456	0.1098
HDF-P25	3.23	198042	42494	1654	300	986	236	56.98%	14.66%	55.90%	13.13%	0.4486	0.0688	0.3616	0.0797	0.7699	0.0613
HDF-P25	3.14	201588	42233	1673	315	972	223	55.79%	14.53%	56.64%	12.50%	0.4529	0.0723	0.3578	0.0774	0.7761	0.0638
HDF-P25	3.42	194620	41142	1689	299	1009	223	54.93%	13.66%	54.54%	12.31%	0.4595	0.0685	0.3752	0.0759	0.7719	0.0612
HDF-P30	3.82	179793	51562	1586	338	890	378	66.65%	13.59%	70.57%	14.30%	0.3760	0.1033	0.2451	0.0816	0.7879	0.0606
HDF-P30	4.41	184842	56571	1576	337	884	367	66.61%	14.38%	69.79%	12.94%	0.3762	0.0993	0.2470	0.0834	0.7862	0.0624
HDF-P30	3.42	178081	51670	1584	326	895	349	66.37%	13.90%	69.63%	12.53%	0.3666	0.0959	0.2468	0.0761	0.7854	0.0590
HDF-P30	3.73	181461	55236	1594	325	911	368	65.97%	14.76%	68.82%	12.22%	0.3837	0.1019	0.2543	0.0763	0.7836	0.0616
HDF-P35	4.45	215455	51384	1603	443	774	397	71.09%	18.21%	73.87%	15.83%	0.3856	0.0990	0.1919	0.0720	0.7764	0.1154
HDF-P35	4.01	213504	49725	1631	439	814	460	70.00%	17.82%	73.23%	17.02%	0.3962	0.0950	0.1961	0.0729	0.7687	0.1187
HDF-P35	4.18	212233	52528	1640	448	768	371	69.31%	18.51%	74.55%	14.86%	0.3994	0.0987	0.1835	0.0654	0.7670	0.1166
HDF-P38	4.61	223879	91231	1388	301	643	205	69.47%	15.11%	79.99%	10.05%	0.3674	0.0757	0.1471	0.0594	0.7689	0.0681
HDF-P38	6.05	226008	89222	1399	315	647	209	68.88%	15.52%	80.15%	10.46%	0.3725	0.0796	0.1487	0.0624	0.7657	0.0701
HDF-P38	4.86	228775	90931	1370	316	650	230	70.57%	15.73%	80.03%	11.08%	0.3612	0.0802	0.1472	0.0648	0.7690	0.0750
HDF-P38	6.08	220862	89690	1390	322	629	217	69.44%	15.95%	81.03%	10.18%	0.3679	0.0815	0.1398	0.0607	0.7672	0.0756

Supplementary File 2

HDF-P43	10.14	247705	87399	1191	251	577	284	80.77%	7.79%	81.12%	11.74%	0.2594	0.0634	0.1329	0.0586	0.7986	0.0801
HDF-SIS	infinity	174841	54741	1320	399	335	175	73.96%	18.17%	87.05%	10.79%	0.3231	0.0970	0.1041	0.0622	0.7874	0.0911
HDF-SIS	infinity	176861	54403	1329	403	336	170	73.04%	18.09%	86.98%	10.62%	0.3264	0.0993	0.1049	0.0610	0.7843	0.0899

Supplementary File 3

For both HDF and DU145 cells, samples treated with 200 μM H_2O_2 reached premature senescence, while majority of cells treated with 500 μM H_2O_2 died after seven days of culture (Tables S3.1 and S3.2). For the control, cell counts at day 7 cannot be accurately reported, as the 6-well culture plate used became completely confluent.

Table S3.1: Cell counts of oxidant-induced premature senescent HDF cells.

Days after Treatment	Control (0 μM)	200 μM	500 μM
-1	400,000	400,000	400,000
0	653,000	336,000	303,000
1	1.031,000	352,000	268,000
7	completely confluent	489,000	187,000

Table S3.2: Cell counts of oxidant-induced premature senescent DU145 cells.

Days after Treatment	Control (0 μM)	200 μM	500 μM
-1	400,000	400,000	400,000
0	676,000	393,000	276,000
1	1,151,000	395,000	249,000
7	completely confluent	421,000	123,000

Supplementary File 4

1. Resolution of confocal microscopy

The resolution of confocal microscopy is primarily dependent upon the wavelength (λ) of the light used and the numerical aperture (NA). For the purposes of this research, DAPI and Alexa

488 fluorophore were used. As previously mentioned, DAPI was excited at 405 nm, while MeC-conjugated Alexa 488 fluorophore was excited at 488 nm. These values were used as λ in the following calculations. For lateral resolution (in *x*, *y* directions), the Rayleigh criterion states that two points are resolved when the first minimum of one Airy disk is aligned with the central maximum of the second Airy disk. According to this definition, the lateral resolution ($r_{lateral}$) is defined as:

$$r_{lateral} = \frac{1.22\lambda}{2(\eta \sin \alpha)} = \frac{1.22\lambda}{2NA} = \frac{0.61\lambda}{NA}$$

where η is the refractive index of medium between the specimen and the objective lens and α is the semi-aperture angle of the objective. If the excitation and emission wavelengths are close to each other, the Airy disk size for confocal microscope is the square of the wide-field (“regular”) microscope Airy disk, and thus the lateral intensity point spread function (PSF) is reduced by about 30 percent. Thus, the lateral resolution in the confocal mode can be approximated as:

$$r_{lateral} = \frac{0.4\lambda}{NA}$$

Thus, the lateral resolution for images used in 3D-qDMI is approximated as 125 nm for DAPI fluorescence and 150 nm for Alexa 488 fluorophore (conjugated to anti-MeC antibody). For high-resolution microscopy using high NA objectives, however, the Rayleigh criterion may not always give meaningful results due to apodization of the amplitude of the marginal (outer) beam. If resolution can be defined as the minimum separation distance at which the two objects can be

sufficiently distinguished, resolution is directly related to the full width at half maximum (FWHM) in intensity PSF. The scalar approximation for FWHM is given as:

$$FWHM_{lateral} = \frac{0.51\lambda}{NA}$$

Using this equation, FWHM is approximated as 160 nm and 191 nm, respectively for DAPI and Alexa 488 fluorophore (conjugated to anti-MeC antibody). To calculate the axial resolution (in *z* direction), a variety of equations can be found in the microscopy literature. One equation commonly used to describe axial resolution is:

$$r_{axial} = \frac{1.4\lambda \cdot \eta}{NA^2}$$

where η is the refractive index of medium between the specimen and the objective lens. The confocal microscopy shown in this section uses 80% glycerol solution, which has η ~ 1.44. Using this equation, the axial resolution is approximated as 487 nm for DAPI fluorescence and 582 nm for Alexa 488 fluorophore (conjugated to anti-MeC antibody).

2. Determining ideal voxel cube size

For topological voxel analysis, the nuclear ROI is partitioned into large number of voxel cubes, with equal size in *x*, *y*, *z* directions (Figure S4.1).

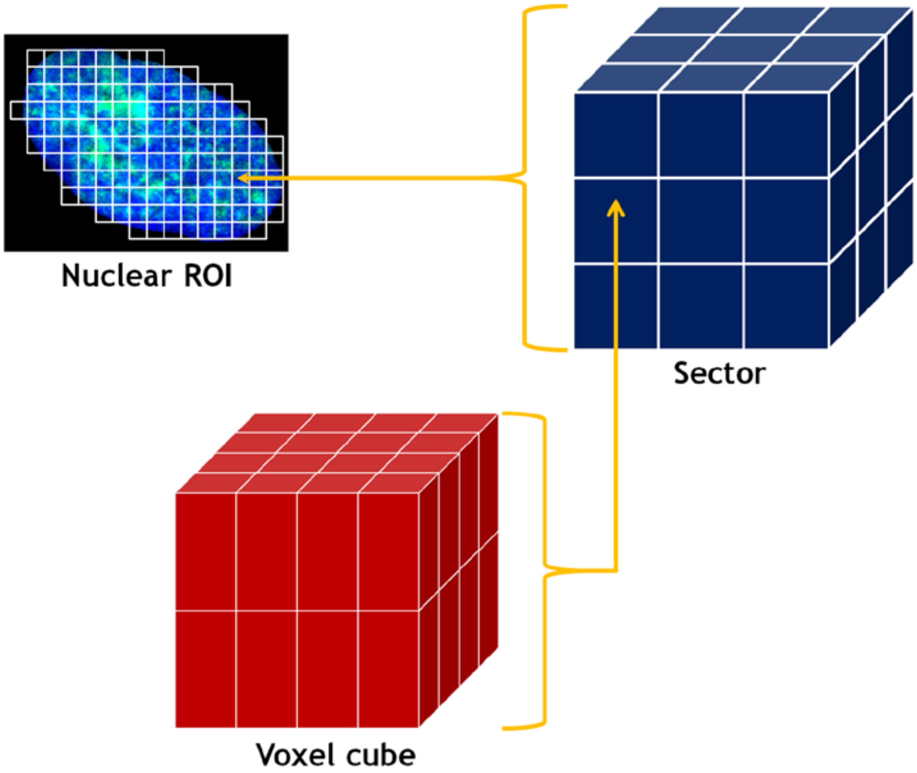

Figure S4.1: Division of nuclear ROI into sub-nuclear compartments. For topological analysis of sub-nuclear features, the ROI is divided into sectors, which are further divided into voxel cubes. Each voxel cube consists of 4(x) x 4(y) x 2(z) pixels, which comprises a physical cube with a side of 500 nm, and each sector is composed of 3(x) x 3(y) x 3(z) voxel cubes.

To determine the size of these cubes, the inherent physical limitations of images need to be considered. As mentioned elsewhere, each pixel in the set of images analyzed for this research has the dimensions of 120 nm (*x*) x 120 nm (*y*) x 250 nm (*z*). In previous section, the axial resolution of the confocal microscopy with the laser scanning confocal microscope (Leica Microsystems TCS SP5X Supercontinuum) and the objective (Leica HCX Plan-Apo 63x, NA=1.3) used for obtaining these images was determined to be around 500 nm. Thus, each voxel cube has the size of 500 nm. Because of the sampling differences in lateral (*x*, *y*) and axial (*z*)

directions, the voxel cube unit was implemented by grouping 4 (x) x 4 (y) x 2 (z) contiguous pixels, 32 pixels in total (Figure S4.2).

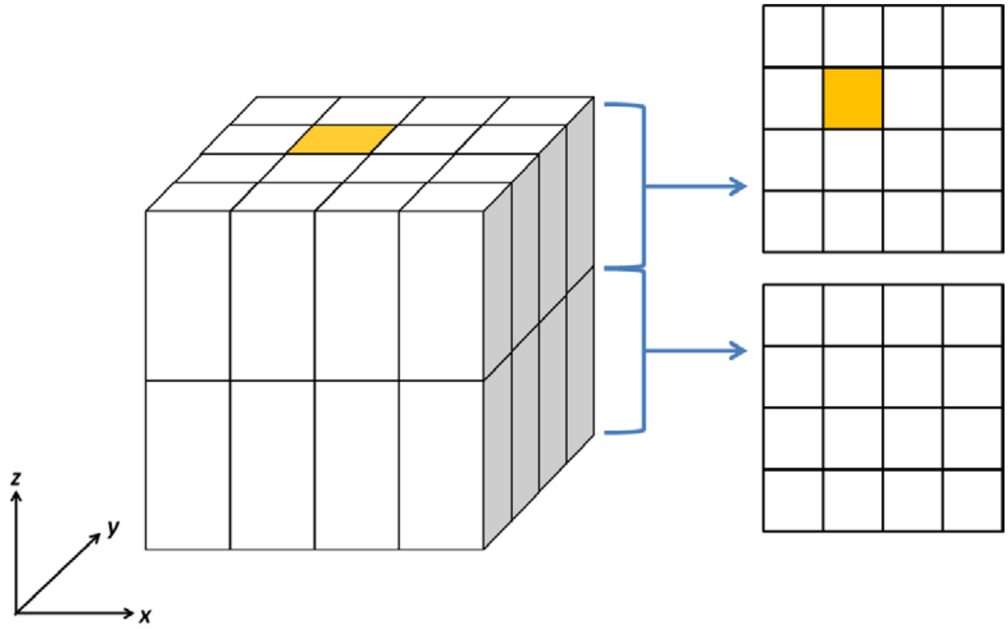

Figure S4.2: Graphical representation of a voxel cube. The voxel cube is defined as 4 (x) x 4 (y) x 2 (z) contiguous pixels, which represent the physical space of 500 nm x 500 nm x 500 nm. A voxel cube consists of maximum 32 pixels.

The actual implementation of splitting nuclear ROIs into voxel cubes occurs by "meshing." For cubes at the edge of these images, in which 4 x 4 x 2 pixels could not be found, extra "blank" pixels were added appropriately so that the size of each voxel cube is uniform. A separate matrix V_{vox} is created to note the number of pixels in each voxel cube that is within the boundaries of nuclear ROI, as defined by the final segmentation map from image pre-processing step of 3D-qDMI.

<u>Supplementary File 5</u>

Prior to any analysis, intensity values of all pixels in nuclear ROI are classified into 3 groups for DAPI channel and 2 groups for MeC channel, based on thresholds $t_{DAPI,low}$, $t_{DAPI,high}$, and t_{MeC}.

The methods for determining these threshold values are shown in Additional File 6. From the thresholds, each pixel is defined as such:

$$I_{DAPI-class}(x,y,z) = \begin{cases} -1, & if\ I_{DAPI}(x,y,z) \leq t_{DAPI,low} \\ 0, & if\ t_{DAPI,low} < I_{DAPI}(x,y,z) < t_{DAPI,high} \\ 1, & if\ I_{DAPI}(x,y,z) \geq t_{DAPI,high} \end{cases}$$

$$I_{MeC-class}(x,y,z) = \begin{cases} 0, & if\ I_{MeC}(x,y,z) \leq t_{MeC} \\ 1, & if\ I_{MeC}(x,y,z) > t_{MeC} \end{cases}$$

Using these classifications, the level of condensation and methylation is calculated from its constituent pixels in the nuclear ROI. The method and parameters used are inspired by a previous publication delineating heterogeneity and condensation of stained chromatin in cell nuclei in two dimensions (Young et al., referenced in [76]) and has been adapted for use with 3-D image processing workflow. To analyze the degree of condensation and methylation and the genomic organization within each voxel cube, the following three parameters are determined: the the condensation level of DNA measured in DAPI channel (*cond*), the total degree of methylation (*meth*), and the associativity of DAPI and MeC channels (*assoc*).

(a) Condensation level of genomic DNA:

To define condensation, two other parameters clumping (*cl*) and charge (*ch*) must be first determined. Clumping (*cl*) measures the spatial localization of intensities and can be defined for either DAPI or MeC channels as:

$$cl_{DAPI} = \frac{\sum_j^{V_{vox}} II_{DAPI}\ (j)}{V_{vox}}, \quad cl_{MeC} = \frac{\sum_j^{V_{vox}} II_{MeC}\ (j)}{V_{vox}}$$

where j is a single pixel in the voxel, V_{vox} is the number of active pixels in the voxel, and II_{DAPI} and II_{MeC} are the integrated intensities in DAPI and MeC channels respectively. Integrated intensity is defined for each pixel in the voxel and is the weighted average of the pixels and its neighbors (Figure S5.1).

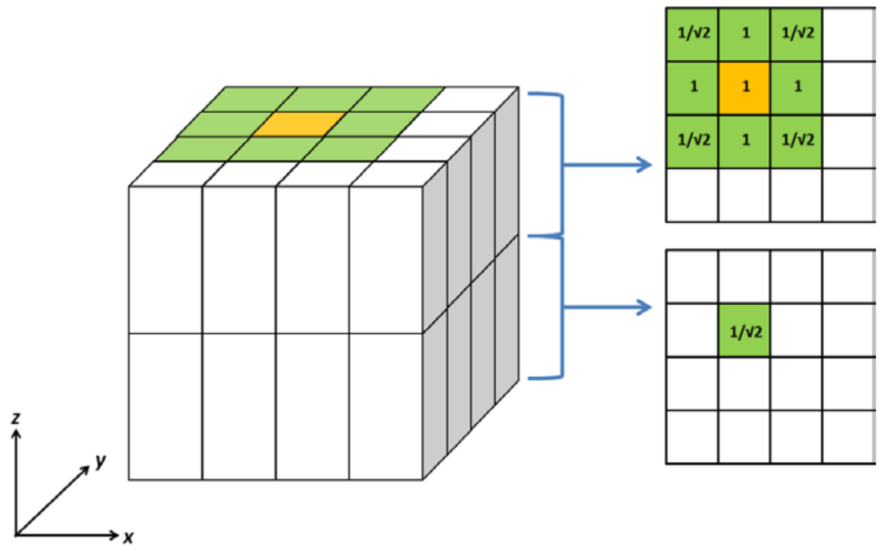

Figure S5.1: Weights used for determining clumping. In calculating clump of a single pixel (shown in gold) for either DAPI or MeC channel, the pixel itself, its 8 neighbors in the same z-plane and its immediate neighbor in the other ¬z-plane is considered. The weights used for each pixel other than itself is determined by the distance from the original pixel to the neighboring pixel.

The weights are set as the inverse of the distance of a pixel to its neighboring pixel. If a pixel does not have a neighbor (limited by the voxel dimensions) or its neighbor is outside the nuclear ROI, the weight for that neighbor is set to zero so that its influence can be neglected. The integrated intensity for pixels in DAPI and MeC channels (II_{DAPI} and II_{MeC} respectively) are defined as:

$$II_{DAPI} = \frac{\sum_i w_i \cdot \left(\frac{I_{DAPI-class}(i) + 1}{2}\right)}{\sum_i w_i}$$

$$II_{MeC} = \frac{\sum_i w_i \cdot \left(I_{MeC-class}(i)\right)}{\sum_i w_i}$$

where *w* is the weight array. Clumping for either channel is restricted to $0 \leq cl \leq 1$. Clumping will give a high value if high-intensity pixels are located next to each other, as its name suggests. Charge (*ch*) reflects the bias towards low- or high-intensity pixels in the DAPI channel and is defined as:

$$ch = \frac{N_{high} - N_{low}}{V_{vox}}$$

With the two prerequisite parameters defined, one can now determine the level of DNA condensation (*cond*) as the normalized product of clumping and charge:

$$cond = k * \frac{\sqrt{|(cl_{DAPI} - 0.5)^2 * ch|}}{2} + 0.5$$

where $k = 1$ if $ch \geq 0$ and $k = -1$ if $ch < 0$. Condensation is constrained $0 \leq cond \leq 1$ and represents the condensation level of the DNA in the voxel. A high condensation level is achieved by large, localized concentration of high-intensity DAPI pixels.

(b) Relative degree of methylation:

The degree of methylation (*meth*) is defined as:

$$meth = \sqrt{cl_{MeC} * comb}$$

where cl_{MeC} is the clumping in the MeC channel previously determined and *comb* is the combined total weight of the two channels, defined as:

$$comb = \frac{\sum_{j}^{V_{vox}} \frac{(I_{DAPI-class}(j) + 2) * I_{MeC-class}(j)}{3}}{V_{vox}}$$

where *j* is a single pixel in the voxel and V_{vox} is the number of active pixels in the voxel. The combined weight is calculated with the assumption that high-intensity pixels in DAPI channels contain more DNA than low-intensity pixels and, thus, more sites are available to be methylated. *comb* is normalized to a value between 0 and 1. By defining *meth* as the geometric mean of cl_{MeC} and *comb*, both the spatial concentration of MeC pixels and the weighted concentration of MeC sites are considered. The actual value of *meth* is constrained to $0 \leq meth \leq 1$.

(c) Associativity of DAPI and MeC channels:

Associativity (*assoc*) determines the correlation of low- or high-intensity pixels in both channels and is defined as:

$$assoc = \frac{\sum_{j}^{V_{vox}} \left(1 - \left(\frac{I_{DAPI-class}(j) + 1}{2} - I_{MeC-class}(j)\right)^{2}\right)}{V_{vox}}$$

where *j* is a single pixel in the voxel and V_{vox} is the number of active pixels in the voxel. The value of *assoc* is constrained to $0 \leq assoc \leq 1$. The closer the two values ($I_{DAPI\text{-}class}$ and $I_{MeC\text{-}class}$) are, the higher the associativity of the voxel is. The three parameters defined above are fairly robust in their ability to distinguish different levels and types of DNA condensation and methylation. The parameters purposely combine intensity information found in both DAPI and MeC channels to derive a more accurate view of nuclear genomic organization and DNA

methylation patterns. Figure S5.2 gives a conceptual model of a voxel cube to show how these parameters would be calculated. These parameters can also highlight very extreme cases. Consider an example in which all 32 pixels in a voxel are represented by high-intensity signals in both DAPI and MeC channels. Then for this example, *cond* = 1, *meth* = 1, and *assoc* = 1, suggesting that this voxel represents an area of very high DNA condensation, as in metaphase chromosomes, with very little variation. At the other extreme, consider a voxel that is entirely composed of low-intensity signals in both channels, representing essentially a "blank" area. In this case, *cond* = 0, *meth* = 0, and *assoc* = 1 (Figure S5.2).

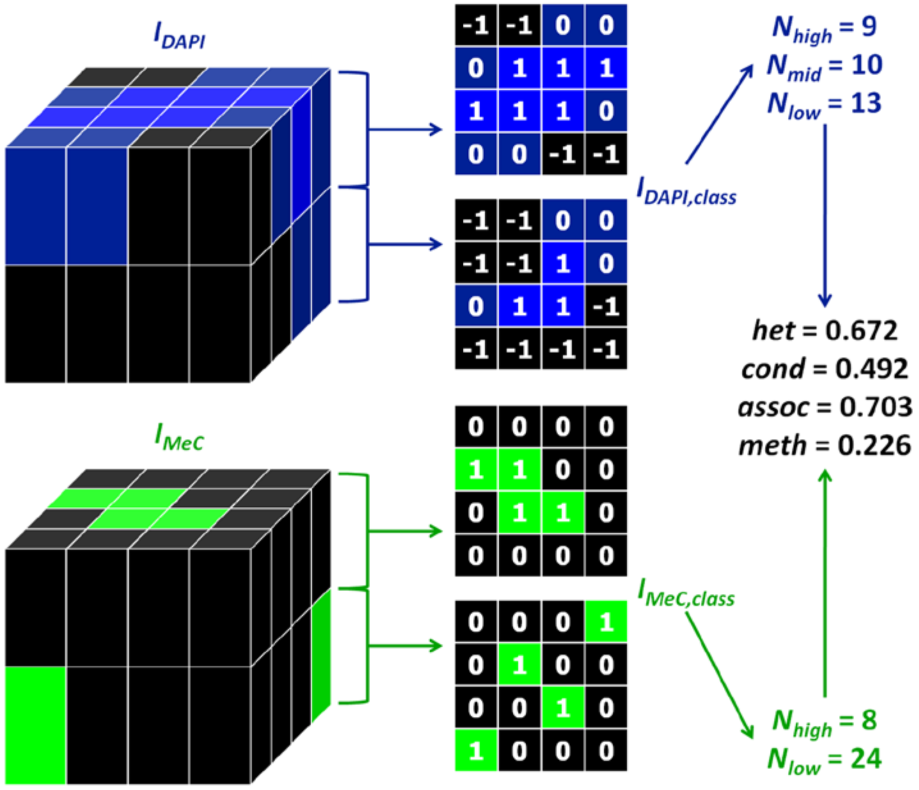

Figure S5.2: Conceptual example of voxel-level analysis of condensation and methylation. A conceptual example of a voxel is shown. From the parameters calculated, this voxel can be inferred as somewhat heterogeneous in DAPI intensity distribution with mid-level DNA

condensation. It is low in methylation, and the MeC sites are highly associated with pixels of high DAPI concentration.

Supplementary File 6

One important criterion needed for topological distribution analysis is the correct classification of pixel intensities as being of low or high intensity. Although all images used for these analyses were acquired under nearly identical conditions and modality settings, even minute changes in these settings could lead to differences in pixel intensity for the same strand of DNA with identical methylation patterns. Thus, classification of pixels into low- and high-intensities should not rely simply on a fixed numerical threshold; instead, the threshold needs to be adjusted based on the inherent characteristics of the image, such as mean background intensity and maximum pixel intensity found in the image. Accurate classification is particularly important for the MeC channel, as low-intensity signals related to hypomethylation or demethylation can be affected by instrument settings (detection PMT voltages, etc.). This issue is less critical in the DAPI channel, since DAPI stains double strand DNA indiscriminately, by intercalating at A-T clusters in the minor groove of the double stranded helix, and, thus, only changes due to instrument settings need to be considered. Therefore, the threshold for each channel is set differently to address these different requirements.

The tripartite classification of pixels in the DAPI channel is performed by introducing two thresholds $t_{DAPI,\,low}$ and $t_{DAPI,\,high}$. These thresholds are determined by:

$$t_{DAPI,low} = k_{D1} \cdot \left(\max(I_{DAPI}) - \mu_{DAPI,back}\right)$$

$$t_{DAPI,high} = k_{D2} \cdot \left(\max(I_{DAPI}) - \mu_{DAPI,back}\right)$$

where $\mu_{DAPI,back}$ is the mean of the background (i.e., all pixels in I_{DAPI} outside nuclear ROIs) found separately during segmentation process, max(I_{DAPI}) is the maximum pixel intensity value found in the ROIs in I_{DAPI}, and k_{D1} and k_{D2} are coefficients determined independently of the specific image analyzed. The coefficients k_{D1} and k_{D2} were determined from I_{DAPI} to account for possible differences in the intensity values among various DAPI channel images, and was obtained prior to any analysis. A large number of randomly chosen images (~ 500) of various cell lines and passage numbers, chosen as a training set. All pixels with nuclear ROIs were then normalized by the mean of the background ($\mu_{DAPI,back}$) and the maximum pixel intensity value found in that image as follows:

$$\overline{I_{DAPI}} = \frac{I_{DAPI} - \mu_{DAPI,back}}{\max(I_{DAPI}) - \mu_{DAPI,back}}$$

The values from all normalized images were then combined to form a 1-dimensional array, which was then sorted. k_{D1} is the 25th percentile value and k_{D2} is the 75th percentile value of this normalized array. The coefficients found for the purposes of this research were k_{D1} = 0.169 and k_{D2} = 0.454.

From the two thresholds, a ternary image $I_{DAPI\text{-}class}$ is defined as follows:

$$I_{DAPI-class}(x,y,z) = \begin{cases} -1, & if\ I_{DAPI}(x,y,z) \leq t_{DAPI,low} \\ 0, & if\ t_{DAPI,low} < I_{DAPI}(x,y,z) < t_{DAPI,high} \\ 1, & if\ I_{DAPI}(x,y,z) \geq t_{DAPI,high} \end{cases}$$

For the MeC channel, as I_{MeC} comes from an antibody staining, the classification procedure is designed to be similar to that of FACS (fluorescence-activated cell sorting): I_{MeC} is separated to regions of MeC^{high} and MeC^{low}. The threshold (t_{MeC}) is determined as follows:

$$t_{MeC} = \max\big(k_M \cdot (\max(I_{MeC}) - \mu_{MeC,back})\big)$$

where $\mu_{MeC,back}$ is the mean of the background (i.e., all pixels in I_{MeC} outside nuclear ROIs) found separately during segmentation process, $\max(I_{MeC})$ is the maximum pixel intensity value found in the ROIs in I_{MeC}, k_M is the coefficient determined independently of the specific image analyzed. The method for finding the coefficient k_M is different from the method used for finding k_{D1} and k_{D2}, since a simple normalization technique yields less than desirable results. As previously mentioned, there is a need to distinguish whether low-intensity signals are caused by instrument settings leading to a generally darker image or the actual absence of MeC in that spatial region. As before, a large number of randomly chosen images (~ 100) of various cell lines and passage numbers were chosen as a training set; however, unlike the method for determining analogous coefficients for I_{DAPI}, masks for nuclear ROIs were not used. Instead, all pixels in I_{MeC}, inside and outside ROIs, from these randomly chosen images were pooled together, without any normalization, to form a large one-dimensional array of pixel intensity values.

Cluster analysis was performed by k-means clustering to separate the array into three clusters. The means of the three clusters will be referred to as μ_1, μ_2, and μ_3. Ideally, the first cluster should represent all pixels from the background, the second cluster should represent low-

intensity pixels, and the third cluster should represent high-intensity pixels. So, k_M is defined as follows:

$$k_M = \frac{\mu_2 - \mu_1}{\text{mean}(\max(I_{MeC})) - \mu_1}$$

where mean(max(I_{MeC})) is the mean of maximum pixel intensity values found in each I_{MeC} of all images used as a training set. By taking the normalized value of μ_2, instead of μ_2 directly, variations due to instrument settings are minimized, as the final threshold t_{MeC} is calculated with characteristics specific to that image. The coefficient k_M found for the purposes of this research was $k_M = 0.176$. From threshold t_{MeC}, a binary image of low-intensity MeC signals I_{LIM} is defined as follows:

$$I_{LIM}(x,y,z) = \begin{cases} 0, & if\ I_{DAPI}(x,y,z) > t_{MeC} \\ 1, & if\ I_{DAPI}(x,y,z) \le t_{MeC} \end{cases}$$
